# Salmon gut microbiota correlates with disease infection status: potential for monitoring health in farmed animals

**DOI:** 10.1186/s42523-021-00096-2

**Published:** 2021-04-20

**Authors:** Davide Bozzi, Jacob A. Rasmussen, Christian Carøe, Harald Sveier, Kristian Nordøy, M. Thomas P. Gilbert, Morten T. Limborg

**Affiliations:** 1 Department of Biotechnology and Biosciences, University of Milano-Bicocca, Milan, Italy; 2 Center for Evolutionary Hologenomics, GLOBE Institute, University of Copenhagen, DK-1353 Copenhagen, Denmark; 3 Laboratory of Genomics and Molecular Medicine, Department of Biology, University of Copenhagen, Copenhagen, Denmark; 4 Lerøy Seafood Group ASA, N-5020 Bergen, Norway; 5 Let Sea AS, 8801 Sandnessjøen, Norway

**Keywords:** Microbiota, Atlantic salmon, Infectious diseases, Dysbiosis, Tenacibaculosis, *Aliivibrio*, *Mycoplasma*, Biomarkers, Fish growth

## Abstract

**Background:**

Infectious diseases cause significant production losses in aquaculture every year. Since the gut microbiota plays an essential role in regulating the host immune system, health and physiology, altered gut microbiota compositions are often associated with a diseased status. However, few studies have examined the association between disease severity and degree of gut dysbiosis, especially when the gut is not the site of the primary infection. Moreover, there is a lack of knowledge on whether bath treatment with formalin, a disinfectant commonly used in aquaculture to treat external infections, might affect the gut microbiome as a consequence of formalin ingestion. Here we investigate, through 16S rRNA gene metabarcoding, changes in the distal gut microbiota composition of a captive-reared cohort of 80 Atlantic salmon (*Salmo salar* L.), in consequence of an external bacterial skin infection due to a natural outbreak and subsequent formalin treatment.

**Results:**

We identified *Tenacibaculum dicentrarchi* as the causative disease pathogen and we show that the distal gut of diseased salmon presented a different composition from that of healthy individuals. A new, yet undescribed, *Mycoplasma* genus characterized the gut of healthy salmon, while in the sick fish we observed an increase in terms of relative abundance of *Aliivibrio* sp., a strain regarded as opportunistic. We also noticed a positive correlation between fish weight and *Mycoplasma* sp. relative abundance, potentially indicating a beneficial effect for its host. Moreover, we observed that the gut microbiota of fish treated with formalin was more similar to those of sick fish than healthy ones.

**Conclusions:**

We conclude that external *Tenacibaculum* infections have the potential of indirectly affecting the host gut microbiota. As such, treatment optimization procedures should account for that. Formalin treatment is not an optimal solution from a holistic perspective, since we observe an altered gut microbiota in the treated fish. We suggest its coupling with a probiotic treatment aimed at re-establishing a healthy community. Lastly, we have observed a positive correlation of *Mycoplasma* sp. with salmon health and weight, therefore we encourage further investigations towards its potential utilization as a biomarker for monitoring health in salmon and potentially other farmed fish species.

**Supplementary Information:**

The online version contains supplementary material available at 10.1186/s42523-021-00096-2.

## Background

The aquaculture market is expanding. As reported by the Food and Agriculture Organization of the United Nations in a 2018 report [1], in 2016 the global aquaculture production was 110.2 million tonnes with a first-sale value of $243.5 Billion (USD).

The world population is assessed at around 7.8 billion people today and an increase of 2 billion is expected by 2050 according to the 2019 United Nations world population prospect (https://population.un.org/wpp/) [2]. As a consequence, despite the remarkable growth of the aquaculture industries, The United Nations Food and Agriculture Organization forecast a global seafood shortage of 50–80 million tonnes by 2030 [1]. As the world population grows, and the demand for seafood increases, the importance of sustainable food production comes with the need to further optimise sustainable farming practices including improved fish health and growth performance.

Fish diseases are a cause of major production losses every year in the world of aquaculture [3] and many of them are caused by bacterial pathogenic infections (e.g. Vibriosis, Furunculosis, Yersiniosis, Tenacibaculosis) [4]. Infectious diseases have been historically treated with the use of antibiotics. However, with the rise of the antibiotic resistance crisis, sustainable alternatives for disease control are gaining momentum [5].

Given the pivotal role played by the fish gut microbiota in regulating host immune system, health status and physiology [6–10], and with an altered gut microbiota often associated with diseases [11–13], interest has risen in the possibility of controlling the fish health status by modulating the gut microbiome through the use of pre-, pro- and synbiotics [14–18].

For the selection of a new probiotic, basic information regarding its genetics and physiology must be gained to better understand its functions and interactions with the other microorganisms in the gut. Identifying the factors that govern the gut microbiota and understanding their effect, is the first step to actively establish and maintain a healthy gut microbiota community [10].

Environment [19], diet [20] and host genotype [21] have been demonstrated to play an important role in defining the Atlantic salmon gut microbiota composition. However, only few studies have examined the association between disease severity and degree of dysbiosis in fish [10, 12, 13]. Diseases caused by external bacterial infections, such as Tenacibaculosis, are responsible for major production losses every year [4], and little is known about potential secondary effects to the gut microbiome during an infection and potential treatment.

Studying disease-induced alterations of the gut microbiota is of crucial importance for understanding the onset and progression of the disease as well as the optimization of the treatment. Studies focusing on analyzing the alteration in the gut microbiota during disease progression could give insight into this process and help to develop new strategies for disease monitoring, prevention and control. As an example, the study of the microbiota in the context of a disease could lead to the identification of microbial gut signatures that correlates with the health status of the fish and may serve as useful biomarkers for monitoring gut health and earlier detections of a disease.

In aquaculture, water disinfection treatments with formalin are commonly applied to treat external infections [3], however, their long-term effect on the fish gut microbiota has, to our knowledge, not been investigated. These treatments, while immediately contrasting the expansion of an external pathogen, can potentially cause an alteration of the healthy gut microbiota composition, and compromise the general fish growth performance by depleting commensal symbionts involved in nutrient metabolism, and overall health status, including immune system modulation.

In the present study, a population of captive-reared Atlantic salmon (*Salmo salar* L.) (approx. one-year-old) was affected by a natural outbreak of an external bacterial infection causing an ulcerative skin disease.

We use 16S rRNA gene metabarcoding to describe the distal gut microbiota of fish affected to different degrees by an external infection as well as the secondary effects of formalin treatment. Our results provide new insight into both disease- and treatment-related alteration of a healthy salmon gut microbiota.

## Methods

### Fish rearing conditions and disease phenotype

The experimental trial was performed at the LetSea land facility (Bjørn, Dønna, Norway) in a seawater based flow-through system heated by heat pump and aerated. Juvenile Atlantic salmon were obtained from the commercial hatchery Grytåga Settefisk AS (Vefsn, Norway). Fish were approximately one-year-old and vaccinated with ALPHA JECT micro® 6 (PHARMAQ), a vaccine that protects against furunculosis, vibriosis, cold water vibriosis, winter sore, and infectious pancreatic necrosis. Salmon were initially kept in brackish water (24 ppt of salinity) for 53 days at 12 °C before being stabilized at 14 °C. The fish were acclimatized whilst being fed commercial feed. Fish were then transferred to 12 replicate 2000 l tanks containing saltwater (33-34 ppt of salinity) directly pumped from the sea and subjected to UV treatment for sterilization. Each tank contained between 200 and 300 fish. After the transfer into saltwater, a subset of the fish in each tank unexpectedly started to develop large skin ulcers (Additional file 1 - Supplementary Figure 1). Some fish showed a more severe ulcer phenotype while others seemed in overall good health condition with no external signs of disease. Fish with ulcers were then considered sick, while fish with no visible wounds were scored as healthy. The lack of visible wounds is not per se proof of the absence of the causal pathogen, but it indicates that fish scored as healthy were at a less progressed stage of the disease. Therefore, we assume that fish scored as healthy were able to resist the pathogen for a longer period serving as a useful reference group of more resilient fish compared to fish clearly affected by the pathogen.

### Diagnostic analysis of disease

To identify the causative agent of the ulcerative disease, a culture-based bacteriological analysis from both wound and kidney swabs samples was performed by Vaxxinova Norway AS. The aim of this analysis was to identify the pathogen and not to describe the composition of the overall microbiome. Two different kinds of culture media were used: marine agar medium and blood agar with 2% NaCl medium. Sequencing of the V1-V2 hypervariable region of the 16S rRNA gene (primers: 27F AGAGTTTGATCCTGGCTCAG; 519R GWATTACCGCGGCKGCTG [22]) was performed for the identification of the bacterial species.

### Water disinfection treatment with formalin

To disinfect the entire water system, formalin was applied (aqueous solution of formaldehyde stabilized with methanol), [23, 24]. For the treatment, we used 1 l of formaldehyde 38% (38 mg/ml) for every 4000 L of water. Formalin was left in the tank water to act for 30 min before reopening the water flow-through. The treatment included two separate disinfection procedures carried out with a period of 4 days in between. Food was withheld for 24 h prior to treatment. Samples were collected before and after the complete formalin treatment and fish mortality was assessed during the trial.

### Distal gut content and distal gut mucosa samples collection

**Fig. 1:**
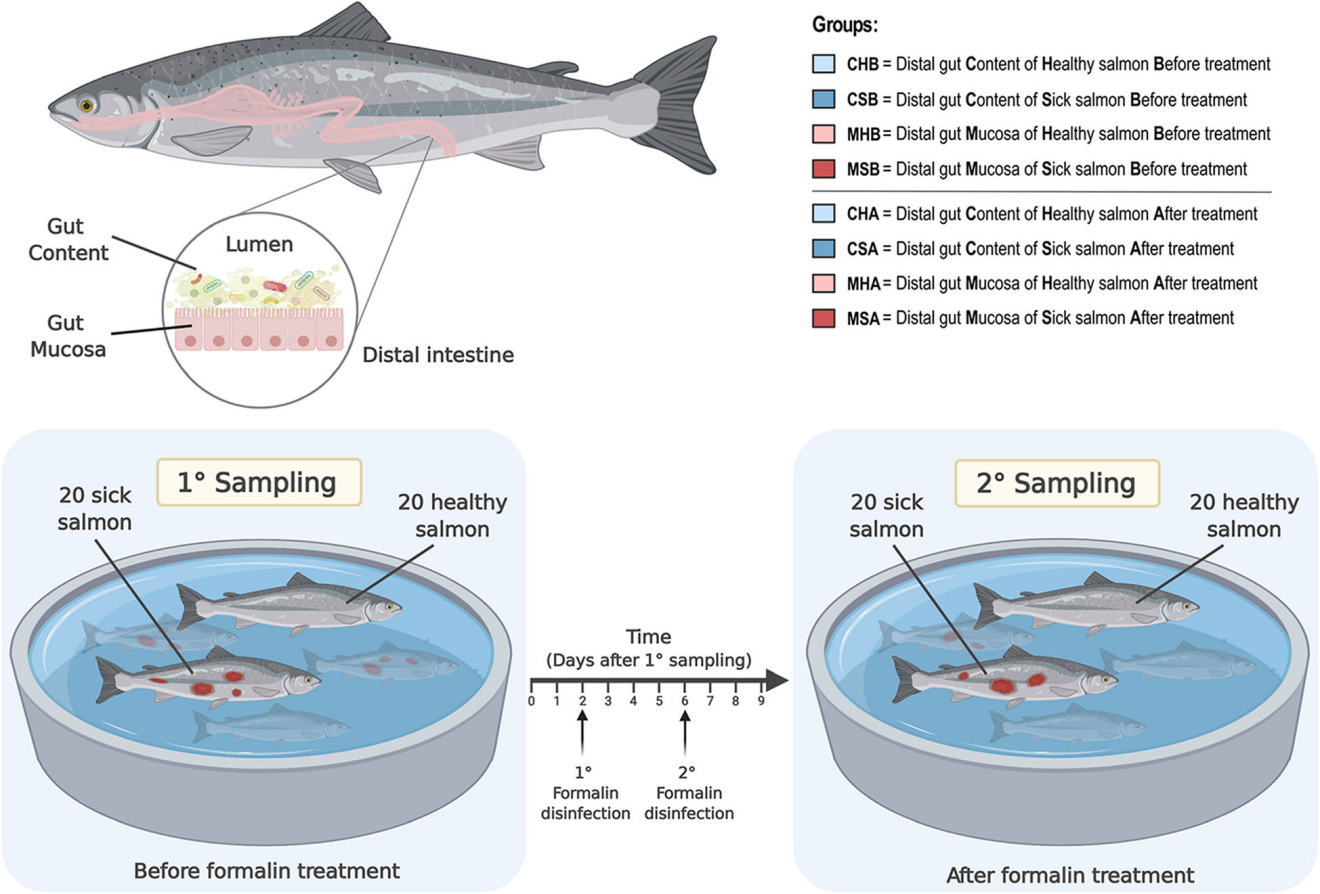
Experimental design and types of samples collected. A total of 80 salmon were sampled, 40 before and 40 after formalin treatment, in both cases, 20 healthy and 20 sick fish were selected (bottom). Two different types of samples, distal gut content and distal gut mucosa, have been collected for each fish (top-left panel), for a total of 160 samples. According to the experimental design for samples collection, eight groups of 20 samples can be recognized as described in the top-right legend: CHB = Distal gut Content of Healthy salmon Before treatment, CSB = Distal gut Content of Sick salmon Before treatment, MHB = Distal gut Mucosa of Healthy salmon Before treatment, MSB = Distal gut Mucosa of Sick salmon Before treatment, CHA = Distal gut Content of Healthy salmon After treatment, CSA = Distal gut Content of Sick salmon After treatment, MHA = Distal gut Mucosa of Healthy salmon After treatment, MSA = Distal gut Mucosa of Sick salmon After treatment. (Created with BioRender.com)

Two sampling events were performed 9 days apart during May 2019: one sampling before and one sampling after formalin treatment. The first sampling was performed 12 days after fish were transferred to saltwater. A total number of 80 fish were sampled from different tanks. Of these, 40 salmon were sampled before formalin treatment and 40 after treatment. At both time points, 20 healthy and 20 sick fish were randomly picked across replicate tanks (Fig. 1), (see Additional file 1 - Supplementary Figure 1 for tank information).

Fish were euthanized using an overdose of Finquel MS-222 (Tricaine Methanesulfonate). Within ca. 10 mins after the euthanization, the abdominal cavity was opened at the ventral midline, and the intestine was aseptically removed. For each salmon, samples from both the distal gut content and the distal gut mucosa were collected (Fig. 1) using scalpels disinfected with bleach 10% and ethanol 70%. For the gut content, we carefully collected content while avoiding the inclusion of host cells, while for the gut mucosa we sampled the intestinal mucosal layer by scraping the epithelial surface using a sterile scalpel. Samples were stored in 1.5 ml sterile Lysing matrix E tubes (MP Biomedicals™) containing 1x DNA/RNA Shield™ buffer (Zymo Research). Tubes were kept at room temperature for transport to the laboratory and then transferred to a − 20 °C freezer until DNA extraction.

Length (cm), weight (g) and gutted weight (g) were measured for each fish to investigate possible correlations between fish dimensions and the gut microbiota composition. Information on the presence or absence of visible wounds were recorded and then utilized to categorize each fish as healthy or sick as defined above.

Three binary variables can be recognized in our experimental design: 1) healthy vs sick fish, 2) before vs after formalin treatment and 3) type of sample (distal gut content vs distal gut mucosa), for a total of eight groups with 20 samples (Fig. 1 and Additional file 2). The above-mentioned groups were used in the subsequent analysis and are hereafter referred to with their acronyms as presented in Fig. 1.

### DNA extraction and quality control

DNA extraction and purification from both distal gut content and distal gut mucosa samples was performed with an in-house extraction protocol as described in the Additional file 3. DNA concentration was assessed with Qubit fluorometric 3.0 quantification (Thermo-Fisher Scientific), following the manufacturer’s recommendations.

Real-time qPCR was performed on all extracts prior to metabarcoding. Specifically, all DNA extracts were pre-screened using SYBR Green qPCR [25] with both primer sets to 1) screen for contamination in extraction negatives, 2) identify the potential presence of PCR inhibitors, and 3) optimise the cycles needed for metabarcoding PCR. The qPCR were performed in 21 μl reactions containing 2 μl DNA template, 9.5 μl of AccuPrime SuperMix II (Invitrogen), 6.5 μl ddH_2_0, 0.5 μM 16S forward primer, 0.5 μM 16S-reverse primer and 1 μl of SYBR Green/ROX solution (Invitrogen). The qPCR amplifications were performed on an Mx3005 qPCR machine (Agilent Technologies) with the following cycling conditions: 95 °C for 10 min, followed by 40 cycles of 95 °C for 30 s, 55 °C for 20 s, and 68 °C for 40 s. Negative controls were included in every qPCR reaction. Using serial dilutions of the DNA template (1:10 and 1:20) we tested for the presence of contaminants (e.g. excess of host DNA), responsible for PCR inhibition [26]. For some samples only the dilutions amplified. In those cases, the 1:10 dilution was selected for the subsequent PCR (Additional file 4).

The Ct values obtained from the qPCR (Additional file 5) were also used to inspect relative differences in the total microbial content of different samples or groups of samples, with the assumption that the 16S rRNA gene can serve as a proxy for the total microbial biomass. We accounted for a potential bias introduced by differences in the 16S rRNA gene copy number among OTUs (see below).

### Metabarcoding and sequencing

For 16S rRNA gene profiling, the following primers were used: 341 F (5′-CCTAYGGGRBGCASCAG-3′) and 806 R (5′-GGACTACNNGGGTATCTAAT-3′), to amplify the V3-V4 region of the 16S rRNA gene [27]. Previous studies have claimed that the V3-V4 region produces more reproducible results and retrieves a greater number of taxa when compared to the V1-V2 region [28, 29]. For this reason, we selected the V3-V4 region to describe the gut microbiota rather than the V1-V2 regions used for the diagnostic analysis described above.

PCR was performed in 0.2 ml PCR tubes using the same reaction composition used for qPCR with the exclusion of the SYBR green and ROX dyes. Tagged primers were used in different combinations to allow multiplexing of samples during sequencing. According to the qPCR results, some distal gut content samples were subjected to 30 PCR cycles of amplification while the others needed 35 PCR cycles to amplify. For the distal gut mucosa samples, some were subjected to 35 PCR cycles while others were given 40 PCR cycles (Additional file 4). Samples from the gut content amplified better than those from the mucosa (see Additional file 5). PCR was performed in triplicates under the following conditions: denaturation at 95 °C for 5 min followed by the determined number of cycles of denaturation at 95 °C for 15 s, annealing at 55 °C for 20 s and extension at 68 °C for 40 s. After the completion of the cycles, samples were left at 68 °C for 10 min for final extension and then cooled to 4 °C. Two PCR blanks were included in all PCR reactions with ultrapure water replacing the DNA template. To reduce the risk of contamination, pre- and post-PCR products were handled in two different laboratories designated for pre-PCR setup and post-PCR processing respectively and PCR master mix solutions were prepared in a designated DNA template-free laboratory. PCR products were visualized using 2% agarose gel electrophoresis (GE) to check the amplification products quality and amount. All the samples in one PCR replicate have been pooled prior to library preparation. To reduce bias introduced by differential amplification between samples, PCR products were pooled at approximately equimolar ratios determined by gel band strength on the agarose gel. Extraction and PCR blanks were included in the pools for downstream quality filtering, but in a non-equimolar fashion to avoid excessive dilution. PCR replicates were purified through SPRI bead purification [30], with a beads-to-sample ratio of 1X, two washing steps in 0.5 ml of ethanol 80% and elution in 35 μl of EB Elution Buffer (10 mM Tris-HCl). DNA concentration measurement was performed with Qubit 3.0 (Thermo-Fisher Scientific), following the manufacturer’s recommendations. We used the Tagsteady protocol [31] to generate seven sequencing libraries, including three PCR replicates from the distal gut content samples, three PCR replicates from the distal gut mucosa samples and one library blank made of ultrapure water. Tagsteady is a PCR-free Illumina library preparation protocol specifically developed for metabarcoding studies to avoid false assignment of sequences to samples [25]. Indexed library quantification was performed using NEBNext® Library Quant Kit for Illumina® (NEB, New England Biolabs), following the manufacturer’s recommendations. Sequencing of 300 bp paired-end reads was performed at the Danish National High-Throughput Sequencing Center, University of Copenhagen, Denmark, using an Illumina MiSeq platform with reagent kit v3, 600 cycles.

### Bioinformatic data processing

Raw reads were quality filtered and de-multiplexed prior to downstream analyses. Read quality was initially checked with FastQC [32]. Sequences were trimmed with AdapterRemoval [33], adapters were removed together with low-quality bases (minquality = 28). Only sequences with a minimum length of 100 bp were retained. AdapterRemoval was also used to merge overlapping paired-end sequences to obtain the entire 16S rRNA gene V3-V4 region covered by the primers. Reads within each amplicon library were demultiplexed and filtered using Begum (https://github.com/shyamsg/Begum), a modified version of DAMe [34]. Singletons were removed and only sequences present in at least two out of three PCR replicates were maintained for downstream analyses. Merged read pairs were further filtered for their length, conserving only sequences with a length between 380 and 480 bp.

The remaining sequences were then used to detect OTUs. OTU clustering was performed with SUMACLUST, using a 97% similarity threshold [35]. The use of higher clustering thresholds can provide an improved resolution and their adoption should always be taken into consideration [36]. As such, we also performed a clustering with a 99% similarity to generate amplicon sequence variants (ASVs). The use of a higher clustering threshold did not increase the resolution of the strains relevant in our study, therefore we opted to use OTUs instead of ASVs to be able to better relate our results with previous studies.

Begum was used to convert the sequences in a suitable format for the clustering and to generate an OTU abundance table from the SUMACLUST output. After the OTU table was generated, sequences were blasted using QIIME (version 1.9.1) [37] against the NCBI nucleotide (nt) database for taxonomy assignment.

### Contaminants identification and removal

We identified and removed sequences originating from putative contaminants. To identify contaminants, all the DNA extraction and PCR amplification negative controls were included in the sequencing. The compositional profile of the negative controls was used to identify putative contaminants. To avoid removal of genuine OTUs present in the negative controls as a consequence of cross-contamination, all OTUs present in the negative controls were further investigated through BLAST search and, in some cases, phylogenetic analysis.

One OTU representing an unknown Mycoplasma was highly dominant in one out of the 11 negative controls while also being dominant in most of the biological samples. For this unknown *Mycoplasma* genus, a maximum likelihood (ML) phylogenetic tree was built. The sequence of the unknown *Mycoplasma* was blasted against NCBI database with blastn. A total of 28 sequences retrieved from GenBank were included in the tree, comprising 15 sequences obtained from fish gut or fish-related samples such as fish farm sediments. *Mycoplasma mobile*, a fish gill pathogen that diverged from all the other sequences, was included as an outgroup. The 29 sequences were aligned with MUSCLE [38] and the multiple alignment was used to build a ML neighbor-joining (NJ) phylogenetic tree with MEGA5 [39]. A model selection test was performed with MEGA5 to test for the optimal substitution model. The General Time Reversible (GTR) substitution model retrieved the lowest BIC scores (Bayesian Information Criterion) and was therefore considered the model that best described the substitution pattern in the data. The ML-NJ-phylogenetic tree was then constructed using the GTR substitution model and 1000 bootstrap tests.

We further investigated a putative non-biological origin of the so-identified contaminants. We combined information from patterns of co-occurrence between the OTUs, their prevalence in samples with low total microbial content and support from previous literature on reagent contamination [40–42]. Spearman correlation coefficient between OTUs (after rarefaction) was calculated in R (version 3.6.3) [43, 44] and plotted as a heatmap with the corrplot R package [45]. The qPCR based Ct values for each sample (Additional file 5) were incorporated in the analysis as a relative proxy for total microbial content to support identified contaminants. Reagent contaminants are known to affect mostly the samples with low total microbial content [40–42, 46]. We visualized this trend by plotting the relative abundances of contaminants (calculated as the sum of all the putative contaminants relative abundances) against Ct values using the “ggscatter” function (ggplot2 R package) [47]. We included OTUs recognized as genuine, OTU1 (*Aliivibrio sp.*) and OTU2 (*Mycoplasma sp.*), for comparison.

We complemented our analysis with a standard method for removing contaminants to avoid unnecessarily strict filtering. Specifically, we applied decontam [48] using the prevalence method as suggested for samples with low biomass and selecting a classification threshold of P* = 0.5.

### Data normalization and diversity analysis

After contaminants removal, also mitochondrial and chloroplast sequences were manually removed. Samples were then normalized by sub-sampling to a depth of 4000 reads. All samples with less than 4000 reads were discarded. It is worth noting that since the rarefaction process may lead to the loss of low abundant OTUs and reduced richness values, studies interested in investigating low abundant OTUs should consider other methods of data normalization [49, 50]. Diversity analyses were conducted, using the hilldiv R-package [51] in Rstudio version 1.2.5033 [43, 44]. Wilcoxon rank-sum test was performed with the hilldiv package to test for statistically significant differences in the mean richness and Shannon index among the described groups.

### Microbial composition analysis

Stacked bar plots representing the microbial composition of the different samples or groups of samples were generated using phyloseq [52], vegan [53] and ggplot2 [47] R packages. We used the gplots package “heatmap.2” function [54] and RColorBrewer [55] to create a heatmap representing OTU abundances among all eight groups. A heatmap dendrogram representing beta diversity among groups was calculated using Jaccard distance metric in vegan. We used Wilcoxon rank-sum test to assess differences in the relative abundance of OTUs among groups of samples. Health status effect and tank effect on the gut microbiota composition were assessed with a permutational analysis of variance (PERMANOVA) using the “adonis” vegan function.

### Correlation between microbiota and fish weight

We investigated potential correlations between the microbiota and the fish weight by calculating the Spearman’s correlation coefficients between fish weight and the relative abundance of the most abundant OTUs (*Aliivibrio* sp. and *Mycoplasma* sp.). For this analysis, only samples before formalin treatment were considered. Spearman correlations were calculated and plotted using ggpubr [56] and ggplot2 R packages. Condition factor K (K value), a normalization of fish weight according to its length, is a useful measure that allows for the standardized assessment of fish condition [57]. We calculated the K value for every fish via the formula: $K=\frac{1{0}^NW}{L^3}$ (where W is the weight of the fish in grams (g), L is the length of the fish in millimeters (mm) and parameter *N* = 5 as suited for salmonids [57]). We then included the calculated K values in the correlation analysis.

Fish were then grouped according to *Aliivibrio* sp. and *Mycoplasma* sp. relative abundances in their microbiota. Salmon with a percentage of *Aliivibrio* sp. higher than 80% (25 fish in total) were pooled together in one group and salmon with a percentage of *Mycoplasma* sp. higher than 80% (37 fish in total) were pooled in another group. All the other samples were discarded from this analysis. After testing for prerequisites, we used Welch’s t-test to assess differences in the mean fish weight between the two groups.

### Comparison of total microbial content

We considered the qPCR Ct values to assess variations in total microbial content among groups. Ct values are inversely proportional to the amount of target DNA in the sample, meaning that in microbial metabarcoding studies the Ct value increases as the amount of total microbial content in the sample decreases. The assumption is that the 16S rRNA gene can serve as a proxy for the actual abundance of microorganisms. A major deviation from this assumption can be introduced by differences in the 16S rRNA gene copy number among microorganisms. We used qPCR Ct values as a proxy for the samples’ total microbial content and tested for differences in the qPCR Ct values among groups with an ANOVA coupled with a Tukey’s HSD post-hoc test for pairwise comparisons in R (version 3.6.3) [43]. We checked that our conclusions were robust by accounting for differences in 16S rRNA gene copy number between *Mycoplasma* sp. and *Aliivibrio* sp., the two OTUs dominating our dataset. We utilized the information on EzBioCloud database (https://www.ezbiocloud.net/) (Accessed 28 July 2020) [58] to estimate the 16S rRNA gene copy number in *Aliivibrio* sp. and *Mycoplasma* sp. Median values for both genera were selected as an approximation of the real copy number value of our OTUs. *Aliivibrio* genus has a median value of 12, while the value for the *Mycoplasma* genus is two. We then calculated a Ct value correction coefficient as log2(12/2) and, for each sample, we multiplied this value for the *Aliivibrio* sp. relative abundance and added the result to the sample Ct value:

$$ Corrected\kern0.5em Ct\kern0.5em value= Ct\kern0.5em value+\left({\mathit{\log}}_2\left(\frac{a}{m}\right)\times Aliivibrio\kern0.5em relative\kern0.5em abundance\right) $$

a = *Aliivibrio* genus median 16S rRNA gene copy number.

m = *Mycoplasma* genus median 16S rRNA gene copy number.

This correction does not account for changes in the OTUs relative abundances as a consequence of the different copy number, which, if included, would reduce the bias by reducing the *Aliivibrio* sp. relative abundance. Therefore, the applied correction is intended as a conservative approach to test the robustness of the Ct value based observations by demonstrating that they are not an artifact derived by differences in 16 rRNA gene copy number.

Data and scripts used for the analysis are available at the GitHub repository: https://github.com/DavideBozzi/Bozzi_et_al_2020_analysis.

## Results

### Identification of the causative disease agent

The bacteriological analysis led to the identification of *Tenacibaculum dicentrarchi* as the most likely causative agent of the ulcerative disease. *Tenacibaculum* is a known pathogenic genus causing ulcerative disease (Tenacibaculosis) in salmonids [59–64]. *Vibrio tapetis*, a known pathogen of cultured clams where it causes brown ring disease [65], was also isolated from the wounds and kidney samples. *V. tapetis* has been previously isolated from diseased fish [66–68] but it is not regarded as a primary pathogen for fish [69].

### Reagent contamination affect samples with lower bacterial biomass

Contamination from the reagent microbiome is a known problem that can affect samples with low bacterial biomass [40–42]. The contaminants identification analysis recognized 22 OTUs as contaminants. The composition of the negative controls was used to identify putative contaminants (Additional file 1 - Supplementary Figure 2). Remarkably, almost all the OTUs found in the negative controls corresponded to well-known reagent contaminants [40–42]. Among all OTUs detected in the negative controls, only OTU2 (an unknown *Mycoplasma* genus) could be clearly assigned, with a phylogenetic analysis, to fish gut or fish-related environments and was therefore retained (Additional file 1 - Supplementary Figure 3). All other OTUs detected as contaminants were discarded. Further support for the non-biological origin of these OTUs comes from the pattern of co-occurrence (Additional file 1 - Supplementary Figure 4), which are known to characterize reagents contaminants [40], and from the observed trend of these OTUs to increase their relative abundance in samples with lower microbial content (higher qPCR Ct value), (Additional file 1 - Supplementary Figure 5). Notably, *Mycoplasma* sp. did not co-occur with the OTUs regarded as contaminants and did not increase its abundance in samples with lower microbial content.

Decontam supported our analysis, recognizing 14 OTUs out of the 22 identified with our customized method as contaminants. The eight OTUs only detected as contaminants in our customized approach were all characterized by having a very low relative abundance, while all the major contaminants were identified by both methods.

### The distal gut microbiota is characterized by low alpha diversity

In total, 13.7 million reads were generated by the Illumina MiSeq sequencing platform for the distal gut content and the distal gut mucosa samples. After quality inspection and trimming, reads were clustered into 130 OTUs using a similarity threshold of 97% (Additional file 6). After taxonomy assignment (Additional file 7), contaminants reads, as well as chloroplasts and mitochondrial reads were removed and the samples were rarefied to 4000 reads per sample. In this way, 47 of the original 160 samples were discarded. For the remaining 113 samples, the contaminants removal procedure and the rarefaction process reduced the total number of OTUs to 65.

The relative abundance of OTUs was highly uneven, with just two OTUs being highly abundant across all samples: *Aliivibrio* sp. and an unknown *Mycoplasma* genus. These two OTUs alone accounted for 99.68% of the total number of reads after filtering and normalization.

BLAST search of the *Aliivibrio* sp. sequence did not identify the specific species since the sequence recovered an exact match (100% of similarity) with two, closely related [70], *Aliivibrio* species: *Aliivibrio logei* and *Aliivibrio salmonicida,* the latter being a well-known pathogen causing cold water vibriosis in salmonids [71].

Conversely, the *Mycoplasma* sp. sequence is part of a yet undescribed genus in the Mycoplasmateceae family. Interestingly, our sequence showed to be phylogenetically related to other *Mycoplasma* species identified in fish gut samples [72–76] which phylogenetically clustered together (Additional file 1 - Supplementary Figure 3), suggesting the existence of a fish-associated *Mycoplasma* genus.

**Fig. 2:**
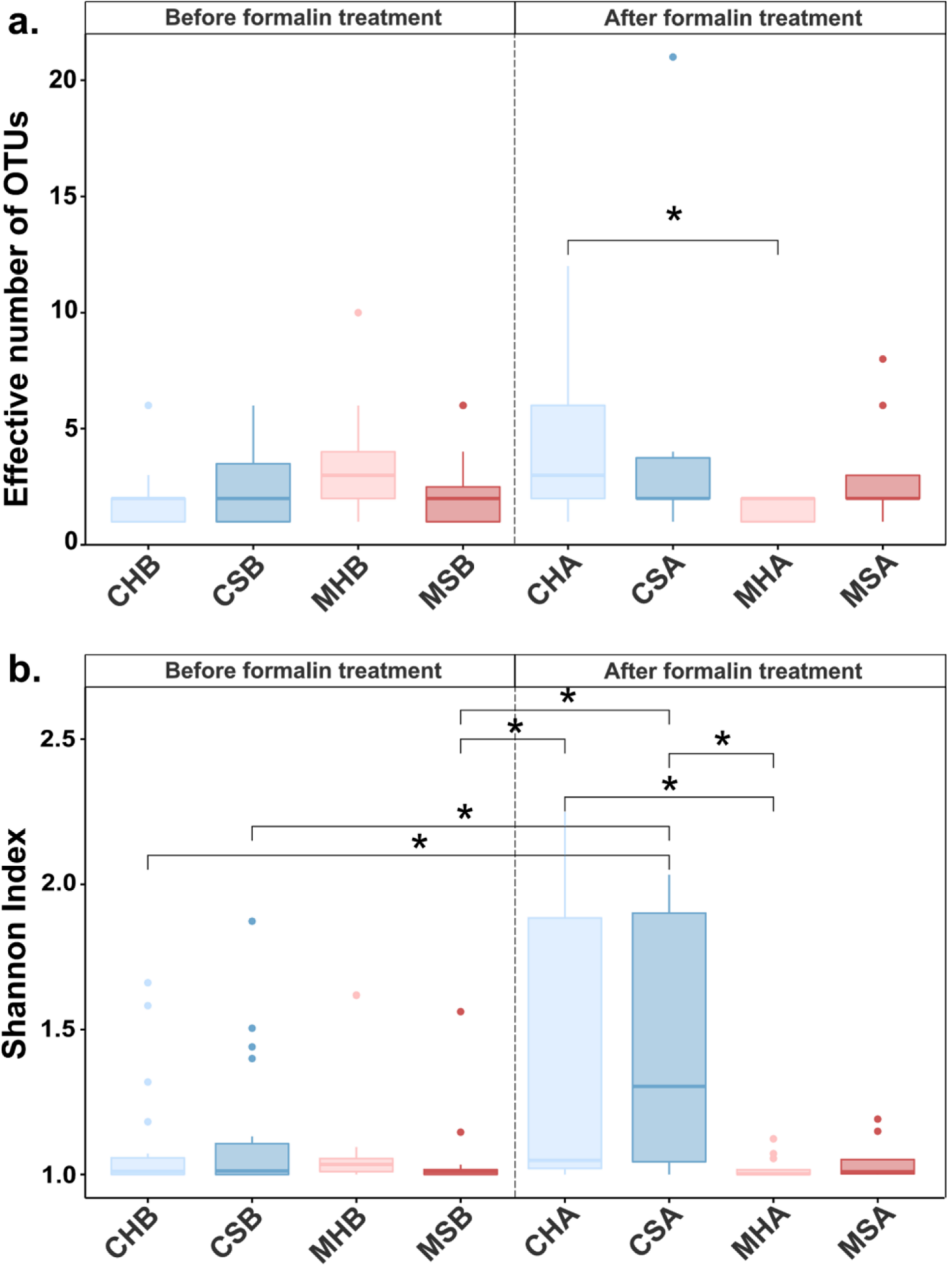
Richness and Shannon index mean values and standard deviations for the eight groups. Boxplot showing the richness (effective number of OTUs) (**a**) and Shannon index (**b**) values for the eight groups. The group name abbreviations are defined in Fig. 1. Groups of samples before formalin treatment are on the left side of the plot, while those after treatment are on the right. Gut content samples are colored in blue while gut mucosa samples are shown in red. Color intensity discriminates between healthy (light) and sick (darker). All groups show low alpha diversities. Statistically significant pairwise differences (Wilcoxon rank-sum test, *p* < 0.05) in the mean richness and Shannon index values among the groups are highlighted with an asterisk (*)

The alpha diversity analysis highlighted a low microbial biodiversity in the investigated salmon intestine (Additional file 8). Samples were clustered according to the eight groups defined in Fig. 1 and mean alpha diversity values for each group are shown in Fig. 2. Given the highly uneven microbial relative abundances, Shannon index values are expected to better describe the system [51]. The Shannon index values highlight a striking feature characterizing the investigated samples: many of them are dominated in the composition by just one OTU (Fig. 2b). The increase of the Shannon index in CHA and CSA indicate the coexistence of the two highly abundant OTUs in some samples of these groups.

### The distal gut microbiota of healthy and sick fish differs in both composition and total microbial content

**Fig. 3:**
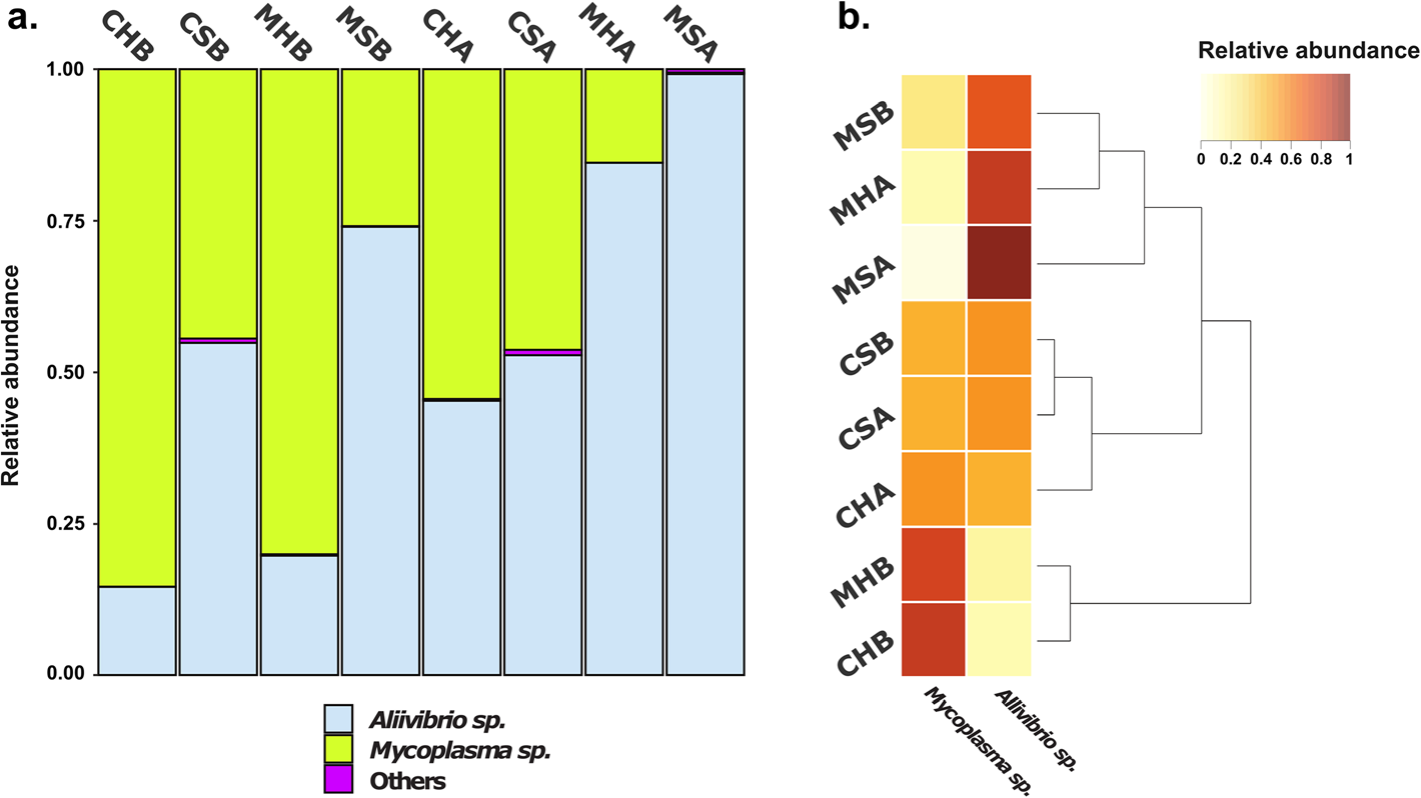
Microbial composition of the investigated groups and groups beta diversity. **a** Barplots depicting the microbial composition of the investigated groups (see Fig. 1 for definition) shows that they are dominated in the composition by two OTUs: *Aliivibrio* sp. and *Mycoplasma* sp. Specific shifts in the relative abundance of the two highly abundant OTUs across groups are visualized. **b** Clustering based on beta diversity (represented as a dendrogram) is mainly determined by the relative abundance of the two dominating OTUs as shown by the heatmap

Relative abundances of the two most abundant bacteria, *Aliivibrio* sp. and *Mycoplasma* sp., have been investigated for both the single samples (Additional file 1 - Supplementary Figure 6) and the eight groups (Fig. 3a). All other OTUs constituted less than 0.5% of the total number of reads and were clustered into a single category referred to as Others. In accordance with the alpha diversity results, many samples were either dominated by *Aliivibrio* sp. or *Mycoplasma* sp. Few samples were characterized by a more even abundance of both OTUs, and many of these samples were from the distal gut content of both healthy and sick fish after formalin treatment. Beta diversity among groups is visualized in Fig. 3b.

A striking difference in the microbiota composition between healthy and diseased salmon was observed. In healthy fish, the relative abundance of *Mycoplasma* sp. was higher in percentage when compared to sick fish and vice versa for *Aliivibrio* sp. (Fig. 3). The observed difference in *Mycoplasma* sp. and *Aliivibrio* sp. relative abundances between healthy and sick fish before formalin treatment was statistically significant (Wilcoxon rank-sum test: *p* < 0.05) and a PERMANOVA analysis showed a statistically significant effect of the fish health status on determining the gut microbiota composition (*R*^2^ = 0.23, *P* ≤ 0.001). Some individual outlier samples were present (Additional file 1 - Supplementary Figure 6). It is possible that fish initially evaluated as healthy were affected by *Tenacibaculum dicentrarchi*, but that they had not developed external ulcers yet.

Beta diversity among groups also showed that CHB and MHB cluster together, indicating that the healthy fish gut microbiota presents a distinct compositional profile that differs from groups affected by the disease and/or the formalin treatment. Together, the Shannon index and the relative abundance observations suggest that the microbiome of healthy fish before formalin treatment is dominated by the unknown *Mycoplasma* sp., while the sick fish gut microbiota is dominated by *Aliivibrio* sp. and that this difference is visible from both the gut content and the gut mucosa samples (Fig. 3).

We observed a statistically significant tank effect on the gut microbiota composition (PERMANOVA *R*^2^ = 0.35, *P* < 0.01). However, since the compositional differences observed between healthy and sick fish were statistically supported (see above), we exclude the possibility of the tank effect having affected our main findings. Instead, we retain that differences in the stage of the disease progression among tanks and discrepancies in the number of healthy and sick fish sampled from different tanks might play a major role in the observed tank effect (Additional file 1 - Supplementary Figure 1).

**Fig. 4:**
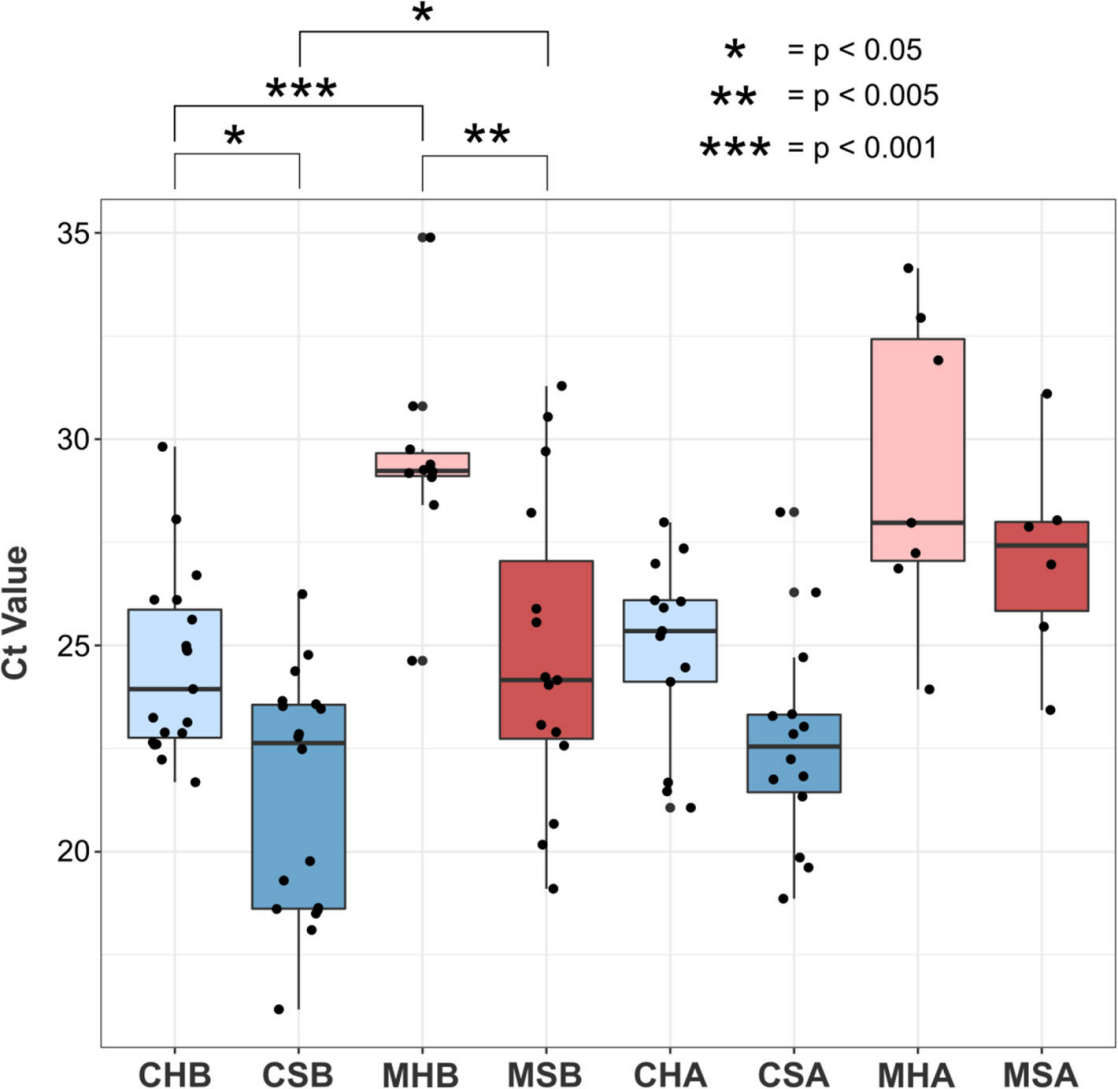
Ct values comparison across groups of samples. The boxplot shows the qPCR Ct values for all sample groups. Groups of samples before formalin treatment are on the left side of the plot, while those after treatment are on the right. Gut content samples are colored in blue while gut mucosa samples in red. Color intensity discriminates between healthy (light) and sick (darker). ANOVA coupled with a Tukey’s HSD post-hoc test for pairwise comparisons was performed. Differences in the mean Ct values across groups can be seen when comparing groups of healthy fish with their sick counterpart (e.g. CHB vs CSB) and when distal gut content groups with their distal gut mucosa counterpart (e.g. CHB vs MHB). Sick fish present lower Ct values than the healthy ones indicating an increase in the total microbial biomass in relation to disease progression, and gut mucosa samples harbor a lower total microbial biomass than the gut content. No difference in the Ct values could be detected in response to formalin treatment indicating a negligible direct effect on the gut total microbial content. Statistically significant differences in the mean Ct value between groups are highlighted for biologically relevant comparisons (see Additional file 9 for all the comparisons *p*-values)

We further investigated possible differences in the total gut microbial content of healthy and sick salmon. Differences in mean Ct values among groups were tested with ANOVA coupled with a Tukey’s HSD post-hoc test for pairwise comparisons (Fig. 4). Statistically significant differences were observed when comparing CHB vs. CSB and MHB vs. MSB. In both cases, the healthy samples presented higher Ct values than their diseased counterpart. This indicates that healthy salmon tend to have lower total microbial content than sick ones, pointing to a disease-associated increase in the total microbial content in the gut.

Taken together, compositional and abundance data indicates that the distal gut microbiota of healthy salmon was colonized almost entirely by *Mycoplasma* sp. Fish affected by the external *T. dicentrarchi* infection experienced an expansion of *Aliivibrio* sp. relative abundance which also corresponded to an increase in the total microbial content in the distal gut.

### High fish weight correlate with a *Mycoplasma* dominated gut microbiota

**Fig. 5:**
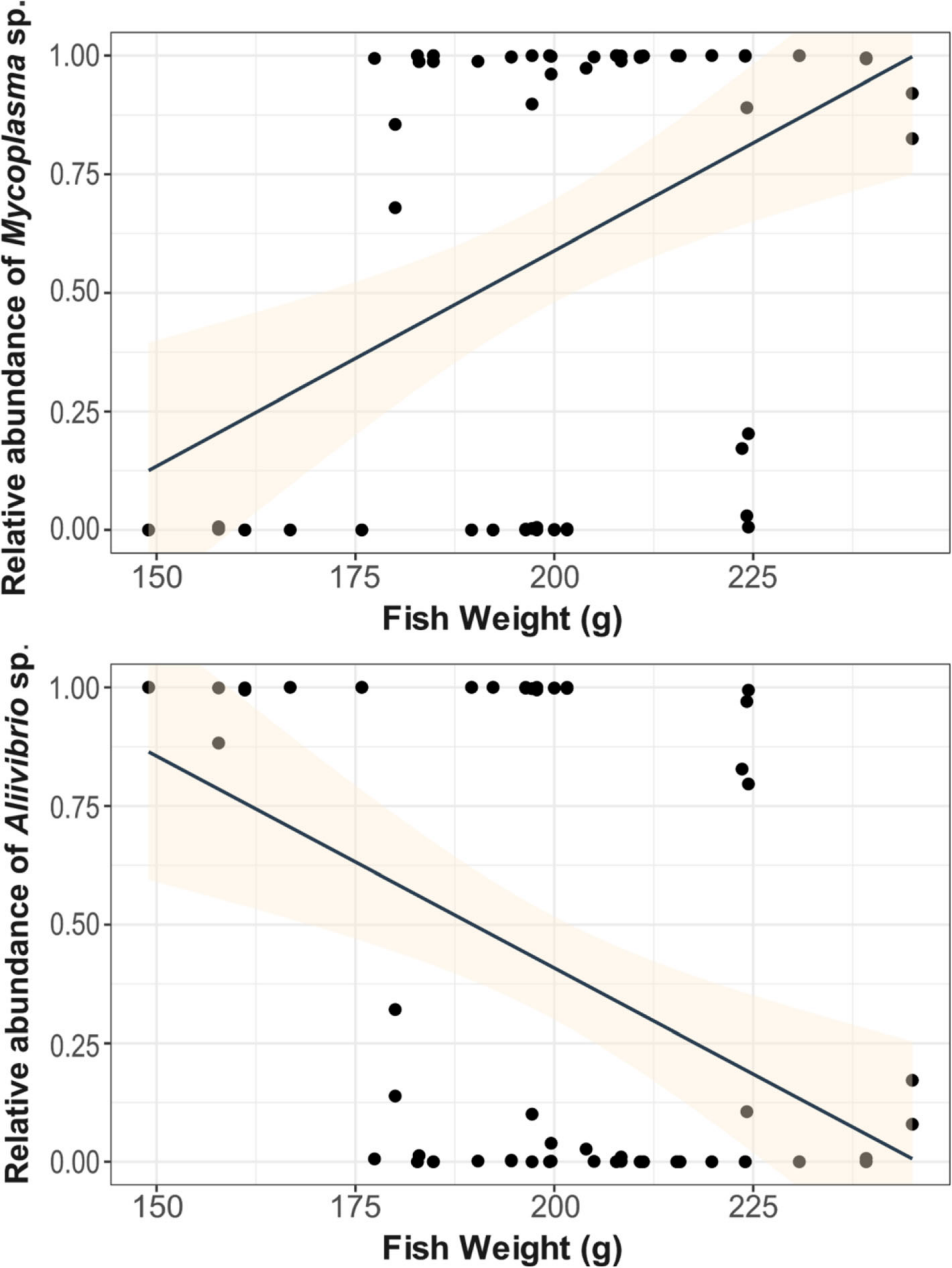
Spearman’s rank correlation between OTUs relative abundance and fish weight. A positive correlation (Spearman’s *R* = 0.43, *p* < 0.001) was found when comparing *Mycoplasma* sp. relative abundance and fish weight (top). Similarly, a negative correlation (Spearman’s *R* = − 0.44, *p* < 0.001) was observed when comparing *Aliivibrio* sp. relative abundance with fish weight (bottom)

We checked the presence of a possible correlation between specific OTUs and other relevant phenotypic traits: fish weight and condition factor K (see methods). Fish weight was positively correlated with *Mycoplasma* sp. relative abundance, and negatively correlated with *Aliivibrio* sp. relative abundance (Fig. 5). Results based on the condition factor K showed a similar pattern (Additional file 1 - Supplementary Figure 7).

Fish with high *Mycoplasma* sp. relative abundance were statistically bigger (mean = 207.6 ± 18.4 g) than fish with high *Aliivibrio* sp. relative abundances (mean = 187.1 ± 22.4 g; Welch’s t-test: *p* < 0.001).

### The distal gut mucosa harbors a lower total microbial biomass than the gut content and is more affected by *Aliivibrio* sp. colonization

Interesting compositional differences were observed between the gut mucosa and gut content microbiota profiles. Mucosa samples have a higher *Aliivibrio* sp. relative abundance compared to the content samples in the groups affected by the disease and/or the formalin treatment (Fig. 3). This spatial difference between sample types indicates that the *Aliivibrio* sp. efficiently colonizes the mucosal tissue, in line with what is expected from pathogenic microorganisms, whose success in the infection largely depends on their ability to adhere to host cells and colonize host tissue [77].

We also observed a signal of different total microbial content between microbiota originating from the gut mucosa and the gut content samples. Statistically significant differences (Tukey’s HSD post-hoc test: *p* < 0.05) were observed in the comparison between the mean Ct values of distal gut content groups and their distal gut mucosa counterparts, with the mucosa always presenting a higher mean Ct value (Fig. 4). This suggests that the distal gut mucosa is characterized by a relatively lower microbial biomass than the distal gut content, an observation in accordance with the fact that only distal gut mucosa samples were affected by reagent contamination. Together, these observations indicate that in some fish the mucosa tissue might have harbored an incredibly low total microbial biomass.

### *Aliivibrio* sp. relative abundance increases after formalin treatment

While formalin treatment proved to induce a reduction in fish mortality (Additional file 1 - Supplementary Figure 8) its effect on the gut microbiome remains debatable. The gut microbiota composition of the formalin treated salmon, both healthy and sick, seems to mimic the composition of the sick fish before treatment, with an increased relative abundance of *Aliivibrio* sp. (Fig. 3a). This increase in *Aliivibrio* sp. relative abundance also in the healthy individuals seems to be responsible for the increase of Shannon index observed in some samples after formalin treatment.

The comparison of the mean Ct values of the groups, before and after formalin treatment, showed no significant differences in the total microbial biomass (Fig. 4). This indicates that the disinfectant was not inducing a reduction in the total gut bacterial biomass of the treated fish.

## Discussion

We identified *Tenacibaculum dicentrarchi* as the most plausible cause of the ulcerative skin disease (Tenacibaculosis). *Vibrio tapetis* was also identified from wound and kidney swabs. *Vibrio tapetis* is expected to be an opportunistic species, taking advantage of the weakened immune system of the host to expand, as previously observed [69]. The disease developed after the juvenile salmon (approximately one-year-old) were moved from freshwater to saltwater, pointing to a possible role of the change in salinity in triggering the Tenacibaculosis, in accordance with previous studies describing changes in fish skin and gut microbiome in response to change in salinity [78–81].

The distal gut microbiota of the investigated salmon was characterized by low levels of alpha diversity compared to previous studies on salmon [12, 19, 82–84]. Almost all fish were characterized by only one, highly abundant OTU, with few samples being characterized by two abundant OTUs. The strict filtering that we applied to the sequence data to minimize the chance of over-inflation of OTU richness from contamination and the rarefaction process could partially explain the low values of richness observed in the salmon distal gut in the present study. However, the low microbial biodiversity observed could be a true biological phenomenon, explained, for example, by the impact of relatively sterile captive rearing conditions in the microbiota ontogeny process. Marine water is highly enriched in pathogens. For this reason, water sterilization procedures are commonly applied in land-based aquaculture systems to control pathogen infections. These practices, while helping to prevent infectious disease outbreaks, may also compromise the colonization of the fish gut by other, potentially beneficial, bacteria. This, combined with a generally more uniform diet, might be responsible for the lower levels of alpha diversity observed in the investigated salmon when compared to their wild counterparts [83].

However, low levels of biodiversity are usually observed also in wild adult salmon, indicating a natural reduction in the microbiome alpha diversity during life-cycle stage progression, with the lowest values observed in marine adults [85]. This suggests that high levels of alpha diversity are not necessarily beneficial in adult salmon in contrast to what is often observed in mammals [86] and that a relatively low microbial biodiversity should be expected also in healthy salmon.

A comparison between healthy and sick fish showed that healthy fish were characterized by higher *Mycoplasma* sp. relative abundances, while sick fish were enriched for *Aliivibrio* sp. This pattern has been previously observed also in salmon infected by *Tenacibaculum finnmarkense* [87]. Differences in the total microbial biomass were found between healthy and sick salmon, with the sick fish characterized by a higher total microbial content. This corresponds to the increase in *Aliivibrio* sp. relative abundance and suggests that *Aliivibrio* sp. might be the driver of the observed increase in the total microbial content in the diseased fish. These observations highlight a correlation between the identified *Aliivibrio* sp. and the disease, pointing to a pathogenic or an opportunistic nature of this strain, as also observed with *Vibrio tapetis* on the skin. Our *Aliivibrio* sp. sequence showed 100% similarity with the salmon pathogen *A. salmonicida*. Interestingly, the studied salmon were vaccinated against *A. salmonicida*, therefore the fish are expected to mount an immune response against this pathogen. The fact that *Aliivibrio* sp. is capable of escaping the host’s immune control might be a consequence of the compromised health condition of the fish affected by the external *T. dicentrarchi* infection. In these conditions, a sick fish may fail to mount an efficient immunological response against *Aliivibrio* sp. These observations suggest that the external skin infection may favor the expansion of other opportunistic species inducing a dysbiosis of the gut microbiota community, potentially predisposing the fish to further infections and compromised health conditions post treatment.

While the formalin treatment is useful to treat the external skin infection, as also supported by the reduced mortality in our salmon cohort after treatment with formalin (Additional file 1 - Supplementary Figure 8), it also induced changes of the gut microbiota community towards profiles similar to those observed in sick fish before treatment. All groups of samples presented higher *Aliivibrio* sp. relative abundances after treatment, which might be the consequence of a disruptive effect exerted by the formalin treatment. Using Ct values as a proxy for microbial biomass revealed that formalin treatment did not induce a reduction in the total gut microbial abundance, although we cannot rule out more subtle effects. Specifically, formalin treatment may have had a larger impact in inhibiting the growth and abundance of *Mycoplasma* sp. compared to the presumed opportunistic/pathogenic *Aliivibrio* sp. in the gut of healthy fish. However, it should also be noted that the absence of an untreated control group at the second time point prevents us from discriminating between direct effects caused by the formalin treatment as opposed to longitudinal changes independent from the treatment itself.

Among the possible confounding factors we exclude a role of salinity in affecting these changes since previous studies have highlighted an increase in *Mycoplasma* sp. abundance in the gut of marine adult salmon [85]. Instead, we retain that the progression of the gut dysbiosis might play a relevant role in the observed changes of the diseased fish gut mucosa microbiota after formalin treatment (MSA). Notably, CSA was the only group of samples that did not show an increase in *Aliivibrio* sp. abundance if compared with CSB, its pre-treatment counterpart. This might be explained by the observed preference of *Aliivibrio* sp. of colonizing the gut mucosa compared to the gut content.

Together, these observations imply that even if formalin is an effective treatment for external infections, it does not avoid the expansion of other opportunistic/pathogenic strains in the gut of diseased fish. Moreover, the formalin treatment seems to compromise the gut of healthy fish. Even after a full recovery from the *T. dicentrarchi* infection, the compromised gut microbiome established during the disease and the formalin treatment might negatively affect the subsequent health status of the fish by making it more prone to develop further infections. In conclusion, it would be advisable to consider strategies, such as probiotic administration, aimed at re-establishing a healthy gut microbiome after formalin treatments.

Compositional and beta diversity results highlighted that gut microbiota in healthy salmon before formalin treatment was almost exclusively characterized by the unknown *Mycoplasma* genus. The Mycoplasmataceae are members of the phylum Tenericutes, class Mollicutes [88] (Note - in the SILVA database, “Tenericutes” has been updated to “Firmicutes” and “Mollicutes” has been updated to “Bacilli.”). They are characterized by small genomes and the absence of a cell wall [88]. Interestingly they are found in a wide range of habitats but each strain seems specifically adapted to a particular host environment as suggested by their reduced genome sizes, which may reflect secondary gene loss after having adapted to the specific niche [88]. This characteristic also makes the Mycoplasmataceae organisms difficult to grow on conventional media. As a consequence, the identification of *Mycoplasma* spp. in salmonids and other fish guts has not been possible using culture-based methods. Since the introduction of culture-free methods for microbiome investigations, such as shotgun metagenomics and targeted gene amplicon sequencing, *Mycoplasma* species have been more often reported in salmonid gut samples, including commercially relevant species such as Atlantic salmon [19, 85, 87, 89–91], Chinook salmon [81, 92], and Rainbow trout [93–97], where *Mycoplasma* spp. often account for the majority of the sequenced reads. Specifically, it has been observed that *Mycoplasma* spp. relative abundances increase during salmon development [98], and in particular after transition to saltwater [85]. In light of these facts, there is a growing interest regarding this microorganism and the functional roles it might play in the gut of salmonids.

The classification of *Mycoplasma* species is not a trivial issue. It has been observed that the *Mycoplasma* genus is a polyphyletic group including species known to be metabolically diverse, with all the species falling into one order (Mycoplasmatales) and one family (Mycoplasmataceae). To address the *Mycoplasma* genus polyphyly issue, Gupta and colleagues [88], have recently proposed the creation of a new order (Mycoplasmoidmales), two new families (Mycoplasmoidaceae fam. Nov. and Metamycoplasmataceae fam. Nov.) and five new genera. Our phylogenetic analysis identified the *Mycoplasma* sp. observed in our study as part of a new, yet undescribed, genus, more closely related to the newly proposed *Malacoplasma* genus [88]. The 16S rRNA gene sequence of the *Mycoplasma* sp. identified in this study clustered with those of other *Mycoplasma* spp. identified in the gut of other fish constituting, a new undescribed genus specific to fish intestines. This new *Mycoplasma* genus might be the result of a long-established symbiosis, in which the microorganisms have evolved to specifically adapt to the fish gut environment [84], as indeed supported by a recent study that, using shotgun metagenomics, suggested that the *Mycoplasma* species dominating the microbiota in healthy salmonids may represent an evolutionary adaptation providing beneficial roles to their host [99]. The lack of the microorganism in the surrounding waters observed by a previous study [100], as well as their small genomes, further support this hypothesis.

The *Mycoplasma* strains found in salmonid gut environments have so far not been associated with any negative fitness effects on their host. This is in contrast to what has been observed in other vertebrates [88], or even with strains found in other fish species [101–103], where *Mycoplasma* species are often pathogenic. In the present study, *Mycoplasma* sp. showed a negative correlation with *Aliivibrio* sp. and characterized the gut microbiota of healthy salmon. Previous studies have reported a similar negative correlation between *Mycoplasma* and pathogenic bacteria like *Flavobacterium psychrophilum* [93], and genera including potentially pathogenic species such as *Aeromonas* spp*.* [97] and *Vibrio* spp. [92]. Together, these observations point to a general trend where *Mycoplasma* sp. can be seen as a biomarker negatively associated with the presence of pathogenic strains.

## Conclusion and perspective

In the present study we have shown how an external bacterial skin infection can cause a systemic shift, favoring the expansion of an opportunistic strain presumably causing dysbiosis of the gut microbiota. Furthermore, fish treated with formalin showed a gut microbiota composition more similar to that of sick fish than to healthy ones. These observations are relevant for treatment optimization, which may include strategies to restore a healthy microbiota profile after infection treatment.

We retain that our results should be considered for the development of a novel 16S rRNA gene barcoding-based monitoring tool. Here, we suggest the possibility of utilizing *Mycoplasma* sp. as a new microbial biomarker to monitor the health status of farmed salmonids in real-time, possibly through non-invasive sampling procedures. The non-invasive sampling of feces has been shown to provide useful information on the fish gut microbiomes [104] and can hence be implemented for such monitoring strategies. If we assume our results represent a general pattern, then temporal monitoring of the relative abundance of the *Mycoplasma* sp. can be used to detect possible pathogen infections earlier than e.g. visual identification of skin ulcers. Such faster diagnostics could allow more timely treatment of the fish before severe phenotypic traits develop, substantially reducing disease-associated production losses.

## Supplementary Information

**Additional file 1.** Supplementary Figures.

**Additional file 2.** Metadata file. File containing all the metadata associated with the samples.

**Additional file 3.** DNA extraction protocol.

**Additional file 4.** PCR info file. File containing the information on the number of PCR cycles and dilution used for each sample.

**Additional file 5.** qPCR Ct values file. File containing the qPCR Ct value for each sample.

**Additional file 6.** Original un-rarefied OTU table with associated OTU sequences.

**Additional file 7.** OTU taxonomy file. Taxonomic classification of each OTU.

**Additional file 8.** Samples alpha diversity.

**Additional file 9. **Table with the *p*-values for all the mean Ct values comparison.

## Data Availability

Sequencing data are provided at the NCBI Sequence Read Archive (SRA) database under the study accession code PRJNA665207. Scripts and data used for the analysis can be found in the Github repository: https://github.com/DavideBozzi/Bozzi_et_al_2020_analysis. Vaxxinova Norway AS diagnosis report is available upon request.

## References

[1] FAO (2018). Meeting the sustainable development goals.

[2] World population prospects 2019. Available at https://population.un.org/wpp/.

[3] Assefa A, Abunna F. Maintenance of fish health in aquaculture: review of epidemiological approaches for prevention and control of infectious disease of fish: Veterinary Medicine International; 2018. https://www.hindawi.com/journals/vmi/2018/5432497/. Accessed 8 Apr 2020. 10.1155/2018/5432497 PMC5846361 29682272

[4] Pridgeon J. Major bacterial diseases in aquaculture and their vaccine development. CAB Rev Perspect Agric Vet Sci Nutr Nat Resour. 2012;7(048):1-16. 10.1079/PAVSNNR20127048.

[5] Ventola CL (2015). The antibiotic resistance crisis. Pharm Ther.

[6] Nayak SK (2010). Role of gastrointestinal microbiota in fish. Aquac Res.

[7] Romero J, Ringø E, Merrifield DL. The gut microbiota of fish. In: Aquaculture nutrition: Wiley; 2014. p. 75–100.

[8] Egerton S, Culloty S, Whooley J, Stanton C, Ross RP. The gut microbiota of marine fish. Front Microbiol. 2018;9:873. 10.3389/fmicb.2018.00873. 10.3389/fmicb.2018.00873 PMC5946678 29780377

[9] de Bruijn I, Liu Y, Wiegertjes GF, Raaijmakers JM (2018). Exploring fish microbial communities to mitigate emerging diseases in aquaculture. FEMS Microbiol Ecol.

[10] Xiong J-B, Nie L, Chen J (2019). Current understanding on the roles of gut microbiota in fish disease and immunity. Zool Res.

[11] Tran NT, Zhang J, Xiong F, Wang G-T, Li W-X, Wu S-G (2018). Altered gut microbiota associated with intestinal disease in grass carp (Ctenopharyngodon idellus). World J Microbiol Biotechnol.

[12] Wang C, Sun G, Li S, Li X, Liu Y (2018). Intestinal microbiota of healthy and unhealthy Atlantic salmon Salmo salar L. in a recirculating aquaculture system. J Oceanol Limnol.

[13] Rosado D, Xavier R, Severino R, Tavares F, Cable J, Pérez-Losada M (2019). Effects of disease, antibiotic treatment and recovery trajectory on the microbiome of farmed seabass ( *Dicentrarchus labrax* ). Sci Rep.

[14] Standen BT, Rawling MD, Davies SJ, Castex M, Foey A, Gioacchini G, Carnevali O, Merrifield DL (2013). Probiotic Pediococcus acidilactici modulates both localised intestinal- and peripheral-immunity in tilapia (Oreochromis niloticus). Fish Shellfish Immunol.

[15] Tarnecki AM, Wafapoor M, Phillips RN, Rhody NR (2019). Benefits of a Bacillus probiotic to larval fish survival and transport stress resistance. Sci Rep.

[16] Nguyen TL (2018). Dietary probiotic effect of lactococcus lactis WFLU12 on low-molecular-weight metabolites and growth of olive flounder (Paralichythys olivaceus). Front Microbiol.

[17] Yi Y, Zhang Z, Zhao F, Liu H, Yu L, Zha J, Wang G (2018). Probiotic potential of Bacillus velezensis JW: antimicrobial activity against fish pathogenic bacteria and immune enhancement effects on Carassius auratus. Fish Shellfish Immunol.

[18] Liu C-H, Wu K, Chu T-W, Wu T-M (2018). Dietary supplementation of probiotic, Bacillus subtilis E20, enhances the growth performance and disease resistance against Vibrio alginolyticus in parrot fish (Oplegnathus fasciatus). Aquac Int.

[19] Dehler CE, Secombes CJ, Martin SAM (2017). Environmental and physiological factors shape the gut microbiota of Atlantic salmon parr (Salmo salar L.). Aquaculture.

[20] Gajardo K (2017). Alternative protein sources in the diet modulate microbiota and functionality in the distal intestine of Atlantic salmon (*Salmo salar*). Appl Environ Microbiol.

[21] Zhang Z, Li D, Xu W, Tang R, Li L (2019). Microbiome of co-cultured fish exhibits host selection and niche differentiation at the organ scale. Front Microbiol.

[22] Turner S, Pryer KM, Miao VP, Palmer JD (1999). Investigating deep phylogenetic relationships among cyanobacteria and plastids by small subunit rRNA sequence analysis. J Eukaryot Microbiol.

[23] Leal JF, Neves MGPMS, Santos EBH, Esteves VI (2018). Use of formalin in intensive aquaculture: properties, application and effects on fish and water quality. Rev Aquac.

[24] Francis-Floyd R (1996). Use of formalin to control fish parasites.

[25] Schnell IB, Bohmann K, Gilbert MTP (2015). Tag jumps illuminated – reducing sequence-to-sample misidentifications in metabarcoding studies. Mol Ecol Resour.

[26] Schrader C, Schielke A, Ellerbroek L, Johne R (2012). PCR inhibitors – occurrence, properties and removal. J Appl Microbiol.

[27] Yu Y, Lee C, Kim J, Hwang S (2005). Group-specific primer and probe sets to detect methanogenic communities using quantitative real-time polymerase chain reaction. Biotechnol Bioeng.

[28] Graspeuntner S, Loeper N, Künzel S, Baines JF, Rupp J (2018). Selection of validated hypervariable regions is crucial in 16S-based microbiota studies of the female genital tract. Sci Rep.

[29] Teng F (2018). Impact of DNA extraction method and targeted 16S-rRNA hypervariable region on oral microbiota profiling. Sci Rep.

[30] DeAngelis MM, Wang DG, Hawkins TL (1995). Solid-phase reversible immobilization for the isolation of PCR products. Nucleic Acids Res.

[31] Carøe C, Bohmann K. Tagsteady: a metabarcoding library preparation protocol to avoid false assignment of sequences to samples. Mol Ecol Resour. 2020. 10.1111/1755-0998.13227. 10.1111/1755-0998.13227 32663358

[32] Andrews S (2010). Babraham bioinformatics - FastQC a quality control tool for high throughput sequence data.

[33] Schubert M, Lindgreen S, Orlando L (2016). AdapterRemoval v2: rapid adapter trimming, identification, and read merging. BMC Res Notes.

[34] Zepeda-Mendoza ML, Bohmann K, Carmona Baez A, Gilbert MTP (2016). DAMe: a toolkit for the initial processing of datasets with PCR replicates of double-tagged amplicons for DNA metabarcoding analyses. BMC Res Notes.

[35] Mercier (2013). SUMATRA and SUMACLUST: fast and exact comparison and clustering of sequences.

[36] Callahan BJ, McMurdie PJ, Holmes SP (2017). Exact sequence variants should replace operational taxonomic units in marker-gene data analysis. ISME J.

[37] Caporaso JG, Kuczynski J, Stombaugh J, Bittinger K, Bushman FD, Costello EK, Fierer N, Peña AG, Goodrich JK, Gordon JI, Huttley GA, Kelley ST, Knights D, Koenig JE, Ley RE, Lozupone CA, McDonald D, Muegge BD, Pirrung M, Reeder J, Sevinsky JR, Turnbaugh PJ, Walters WA, Widmann J, Yatsunenko T, Zaneveld J, Knight R (2010). QIIME allows analysis of high-throughput community sequencing data. Nat Methods.

[38] Edgar RC (2004). MUSCLE: multiple sequence alignment with high accuracy and high throughput. Nucleic Acids Res.

[39] Tamura K, Peterson D, Peterson N, Stecher G, Nei M, Kumar S (2011). MEGA5: molecular evolutionary genetics analysis using maximum likelihood, evolutionary distance, and maximum parsimony methods. Mol Biol Evol.

[40] de Goffau MC (2018). Recognizing the reagent microbiome. Nat Microbiol.

[41] Salter SJ (2014). Reagent and laboratory contamination can critically impact sequence-based microbiome analyses. BMC Biol.

[42] Glassing A, Dowd SE, Galandiuk S, Davis B, Chiodini RJ (2016). Inherent bacterial DNA contamination of extraction and sequencing reagents may affect interpretation of microbiota in low bacterial biomass samples. Gut Pathog.

[43] R Core Team and R Foundation for Statistical Computing (2020). R: a language and environment for statistical computing.

[44] RStudio Team (2020). RStudio: integrated development for R. RStudio, PBC, Boston, MA.

[45] Wei T, Simko V (2017). R package “corrplot”: visualization of a correlation matrix (version 0.84).

[46] Dahlberg J (2019). Microbiota data from low biomass milk samples is markedly affected by laboratory and reagent contamination. PLoS ONE.

[47] Wickham H. ggplot2: elegant graphics for data analysis, 978–3–319-24277-4. Springer-Verlag; 2016. https://ggplot2.tidyverse.org.

[48] Davis NM, Proctor DM, Holmes SP, Relman DA, Callahan BJ. Simple statistical identification and removal of contaminant sequences in marker-gene and metagenomics data. Microbiome. 2018;6(1):226. 10.1186/s40168-018-0605-2. 10.1186/s40168-018-0605-2 PMC6298009 30558668

[49] Gloor GB, Macklaim JM, Pawlowsky-Glahn V, Egozcue JJ. Microbiome datasets are compositional: and this is not optional. Front Microbiol. 2017;8:2224. 10.3389/fmicb.2017.02224. 10.3389/fmicb.2017.02224 PMC5695134 29187837

[50] Weiss S, et al. Normalization and microbial differential abundance strategies depend upon data characteristics. Microbiome. 2017;5(1):27. 10.1186/s40168-017-0237-y. 10.1186/s40168-017-0237-y PMC5335496 28253908

[51] Alberdi A, Gilbert MTP (2019). A guide to the application of hill numbers to DNA-based diversity analyses. Mol Ecol Resour.

[52] McMurdie PJ, Holmes S (2013). phyloseq: an R package for reproducible interactive analysis and graphics of microbiome census data. PLoS ONE.

[53] Oksanen J, Blanchet FG, Friendly M, Kindt R, Legendre P, McGlinn D, Minchin PR, O’Hara RB, Simpson GL, Solymos P, Stevens MHH, Szoecs E, Wagner H (2019). vegan: community ecology package.

[54] Warnes GR, Bolker B, Bonebakker L, Gentleman R, Huber W, Liaw A, Lumley T, Maechler M, Magnusson A, Moeller S, Schwartz M, Venables B (2020). gplots: various R programming tools for plotting data.

[55] Neuwirth E (2014). RColorBrewer: ColorBrewer palettes.

[56] Kassambara A (2020). ggpubr: “ggplot2” based publication ready plots.

[57] Barnham C, Baxter A (2003). Condition factor, K, for salmonid fish.

[58] Yoon S-H, Ha SM, Kwon S, Lim J, Kim Y, Seo H, Chun J (2017). Introducing EzBioCloud: a taxonomically united database of 16S rRNA gene sequences and whole-genome assemblies. Int J Syst Evol Microbiol.

[59] Avendaño-Herrera R, Toranzo AE, Magariños B (2006). Tenacibaculosis infection in marine fish caused by Tenacibaculum maritimum: a review. Dis Aquat Org.

[60] Pérez-Pascual D, et al. The complete genome sequence of the fish pathogen Tenacibaculum maritimum provides insights into virulence mechanisms. Front Microbiol. 2017;8:1542. 10.3389/fmicb.2017.01542. 10.3389/fmicb.2017.01542 PMC5561996 28861057

[61] Småge SB, Brevik ØJ, Duesund H, Ottem KF, Watanabe K, Nylund A (2016). Tenacibaculum finnmarkense sp. nov., a fish pathogenic bacterium of the family Flavobacteriaceae isolated from Atlantic salmon. Antonie Van Leeuwenhoek.

[62] Avendaño-Herrera R, Irgang R, Sandoval C, Moreno-Lira P, Houel A, Duchaud E, Poblete-Morales M, Nicolas P, Ilardi P (2016). Isolation, characterization and virulence potential of Tenacibaculum dicentrarchi in salmonid cultures in Chile. Transbound Emerg Dis.

[63] Grothusen H, et al. First complete genome sequence of Tenacibaculum dicentrarchi, an emerging bacterial pathogen of salmonids. Genome Announc. 2016;4(1):e01756–15. 10.1128/genomeA.01756-15. 10.1128/genomeA.01756-15 PMC4759079 26893432

[64] Klakegg Ø, Abayneh T, Fauske AK, Fülberth M, Sørum H (2019). An outbreak of acute disease and mortality in Atlantic salmon (Salmo salar) post-smolts in Norway caused by Tenacibaculum dicentrarchi. J Fish Dis.

[65] Borrego JJ (1996). Vibrio tapetis sp. nov., the causative agent of the brown ring disease affecting cultured clams. Int J Syst Evol Microbiol.

[66] Reid HI, Duncan HL, Laidler LA, Hunter D, Birkbeck TH (2003). Isolation of Vibrio tapetis from cultivated Atlantic halibut (Hippoglossus hippoglossus L.). Aquaculture.

[67] Jensen S, Samuelsen OB, Andersen K, Torkildsen L, Lambert C, Choquet G, Paillard C, Bergh Ø (2003). Characterization of strains of Vibrio splendidus and V. tapetis isolated from corkwing wrasse Symphodus melops suffering vibriosis. Dis Aquat Org.

[68] Declercq AM, Chiers K, Soetaert M, Lasa A, Romalde JL, Polet H, Haesebrouck F, Decostere A (2015). Vibrio tapetis isolated from vesicular skin lesions in Dover sole Solea solea. Dis Aquat Org.

[69] Bergh Ø, Samuelsen OB (2007). Susceptibility of corkwing wrasse Symphodus melops, goldsinny wrasse Ctenolabrus rupestis, and Atlantic salmon Salmo salar smolt, to experimental challenge with Vibrio tapetis and Vibrio splendidus isolated from corkwing wrasse. Aquac Int.

[70] Urbanczyk H, Ast JC, Higgins MJ, Carson J, Dunlap PV (2007). Reclassification of Vibrio fischeri, Vibrio logei, Vibrio salmonicida and Vibrio wodanis as Aliivibrio fischeri gen. nov., comb. nov., Aliivibrio logei comb. nov., Aliivibrio salmonicida comb. nov. and Aliivibrio wodanis comb. nov.. Int J Syst Evol Microbiol.

[71] Kashulin A, Seredkina N, Sørum H (2017). Cold-water vibriosis. The current status of knowledge. J Fish Dis.

[72] Bano N, Smith AD, Bennett W, Vasquez L, Hollibaugh JT (2007). Dominance of mycoplasma in the guts of the long-jawed Mudsucker, Gillichthys mirabilis, from five California salt marshes. Environ Microbiol.

[73] Kim D-H, Brunt J, Austin B (2007). Microbial diversity of intestinal contents and mucus in rainbow trout (Oncorhynchus mykiss). J Appl Microbiol.

[74] Tamminen M, Karkman A, Corander J, Paulin L, Virta M (2011). Differences in bacterial community composition in Baltic Sea sediment in response to fish farming. Aquaculture.

[75] Green TJ, Smullen R, Barnes AC (2013). Dietary soybean protein concentrate-induced intestinal disorder in marine farmed Atlantic salmon, Salmo salar is associated with alterations in gut microbiota. Vet Microbiol.

[76] Xing M, Hou Z, Yuan J, Liu Y, Qu Y, Liu B (2013). Taxonomic and functional metagenomic profiling of gastrointestinal tract microbiome of the farmed adult turbot (Scophthalmus maximus). FEMS Microbiol Ecol.

[77] Pizarro-Cerdá J, Cossart P (2006). Bacterial adhesion and entry into host cells. Cell.

[78] Lokesh J, Kiron V (2016). Transition from freshwater to seawater reshapes the skin-associated microbiota of Atlantic salmon. Sci Rep.

[79] Schmidt VT, Smith KF, Melvin DW, Amaral-Zettler LA (2015). Community assembly of a euryhaline fish microbiome during salinity acclimation. Mol Ecol.

[80] Zhang M, Sun Y, Liu Y, Qiao F, Chen L, Liu WT, du Z, Li E (2016). Response of gut microbiota to salinity change in two euryhaline aquatic animals with reverse salinity preference. Aquaculture.

[81] Zhao R, Symonds JE, Walker SP, Steiner K, Carter CG, Bowman JP, Nowak BF (2020). Salinity and fish age affect the gut microbiota of farmed Chinook salmon (Oncorhynchus tshawytscha). Aquaculture.

[82] Fogarty C, Burgess CM, Cotter PD, Cabrera-Rubio R, Whyte P, Smyth C, Bolton DJ (2019). Diversity and composition of the gut microbiota of Atlantic salmon (Salmo salar) farmed in Irish waters. J Appl Microbiol.

[83] Webster TMU, Rodriguez-Barreto D, Castaldo G, Gough P, Consuegra S, de Leaniz CG (2020). Environmental plasticity and colonisation history in the Atlantic salmon microbiome: a translocation experiment. Mol Ecol.

[84] Heys C (2020). Neutral processes dominate microbial community assembly in atlantic salmon, *Salmo salar*. Appl Environ Microbiol.

[85] Llewellyn MS (2016). The biogeography of the atlantic salmon ( *Salmo salar* ) gut microbiome. ISME J.

[86] Yatsunenko T, Rey FE, Manary MJ, Trehan I, Dominguez-Bello MG, Contreras M, Magris M, Hidalgo G, Baldassano RN, Anokhin AP, Heath AC, Warner B, Reeder J, Kuczynski J, Caporaso JG, Lozupone CA, Lauber C, Clemente JC, Knights D, Knight R, Gordon JI (2012). Human gut microbiome viewed across age and geography. Nature.

[87] Karlsen C, Ottem KF, Brevik ØJ, Davey M, Sørum H, Winther-Larsen HC (2017). The environmental and host-associated bacterial microbiota of Arctic seawater-farmed Atlantic salmon with ulcerative disorders. J Fish Dis.

[88] Gupta RS, Sawnani S, Adeolu M, Alnajar S, Oren A (2018). Phylogenetic framework for the phylum Tenericutes based on genome sequence data: proposal for the creation of a new order Mycoplasmoidales Ord. Nov., containing two new families Mycoplasmoidaceae fam. Nov. and Metamycoplasmataceae fam. Nov. harbouring Eperythrozoon, Ureaplasma and five novel genera. Antonie Van Leeuwenhoek.

[89] Holben WE, Williams P, Saarinen M, Särkilahti LK, Apajalahti JHA (2002). Phylogenetic analysis of intestinal microflora indicates a novel mycoplasma phylotype in farmed and wild salmon. Microb Ecol.

[90] Zarkasi KZ, Abell GCJ, Taylor RS, Neuman C, Hatje E, Tamplin ML, Katouli M, Bowman JP (2014). Pyrosequencing-based characterization of gastrointestinal bacteria of Atlantic salmon (Salmo salar L.) within a commercial mariculture system. J Appl Microbiol.

[91] Zarkasi KZ, Taylor RS, Abell GCJ, Tamplin ML, Glencross BD, Bowman JP (2016). Atlantic Salmon (Salmo salar L.) gastrointestinal microbial community dynamics in relation to digesta properties and diet. Microb Ecol.

[92] Ciric M, Waite D, Draper J, Jones JB (2019). Characterization of mid-intestinal microbiota of farmed Chinook salmon using 16S rRNA gene metabarcoding. Arch Biol Sci.

[93] Brown RM, Wiens GD, Salinas I (2019). Analysis of the gut and gill microbiome of resistant and susceptible lines of rainbow trout (Oncorhynchus mykiss). Fish Shellfish Immunol.

[94] Lowrey L, Woodhams DC, Tacchi L, Salinas I (2015). Topographical mapping of the rainbow trout (Oncorhynchus mykiss) microbiome reveals a diverse bacterial community with antifungal properties in the skin. Appl Environ Microbiol.

[95] Lyons PP, Turnbull JF, Dawson KA, Crumlish M (2017). Phylogenetic and functional characterization of the distal intestinal microbiome of rainbow trout Oncorhynchus mykiss from both farm and aquarium settings. J Appl Microbiol.

[96] Lyons PP, Turnbull JF, Dawson KA, Crumlish M (2017). Effects of low-level dietary microalgae supplementation on the distal intestinal microbiome of farmed rainbow trout Oncorhynchus mykiss (Walbaum). Aquac Res.

[97] Rimoldi S, Gini E, Iannini F, Gasco L, Terova G. The effects of dietary insect meal from *Hermetia illucens* prepupae on autochthonous gut microbiota of rainbow trout (*Oncorhynchus mykiss*). Anim Open Access J MDPI. 2019;9(4):143. 10.3390/ani9040143. 10.3390/ani9040143 PMC6523354 30987067

[98] Minich JJ, et al. Microbial ecology of atlantic salmon (*Salmo salar*) hatcheries: impacts of the built environment on fish mucosal microbiota. Appl Environ Microbiol. 2020;86(12):e00411-20. 10.1128/AEM.00411-20. 10.1128/AEM.00411-20 PMC7267192 32303543

[99] Rasmussen JA, et al. Genome-resolved metagenomics suggests a mutualistic relationship between *Mycoplasma* and salmonid hosts. 2021, PREPRINT (Version 1) available at Research Square 10.21203/rs.3.rs-269923/v1 10.1038/s42003-021-02105-1 PMC8121932 33990699

[100] Webster TMU, Consuegra S, Hitchings M, de Leaniz CG. Interpopulation variation in the atlantic salmon microbiome reflects environmental and genetic diversity. Appl Environ Microbiol. 2018;84(16):1-14. 10.1128/AEM.00691-18. 10.1128/AEM.00691-18 PMC6070748 29915104

[101] Legrand TPRA, Catalano SR, Wos-Oxley ML, Wynne JW, Weyrich LS, Oxley APA (2020). Antibiotic-induced alterations and repopulation dynamics of yellowtail kingfish microbiota. Anim Microbiome.

[102] Legrand TPRA, Wynne JW, Weyrich LS, Oxley APA (2020). Investigating both mucosal immunity and microbiota in response to gut enteritis in yellowtail kingfish. Microorganisms.

[103] Gaulke CA (2019). A longitudinal assessment of host-microbe-parasite interactions resolves the zebrafish gut microbiome’s link to Pseudocapillaria tomentosa infection and pathology. Microbiome.

[104] Anslan S, Li H, Künzel S, Vences M. Microbiomes from feces *vs.* gut in aquatic vertebrates: distinct community compositions between substrates and preservation methods. Microbiology. 2019. 10.1101/651612.

