# Supplementary material for: Salmon gut microbiota correlates with disease infection status: potential for monitoring health in farmed animals
Source: Anim Microbiome. 2021 Apr 20;3:30. doi: 10.1186/s42523-021-00096-2 (PMC8056536; doi:10.1186/s42523-021-00096-2)
Supplement: Supplementary file 1 — Additional file 1. Supplementary Figures. [file 42523_2021_96_MOESM1_ESM.pdf]

Additional File 1 - Supplementary Figures

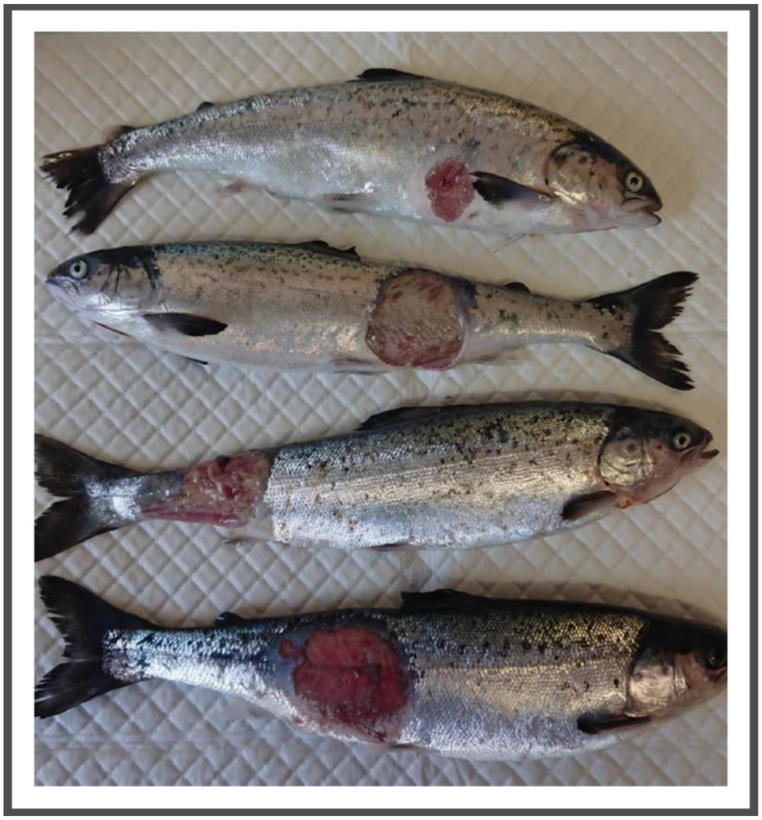

Number of fish sampled from each tank and associated heath status

Tank Number	Healthy Fish	Sick Fish	
T1	1	1	Before Treatment
T3	2	2	
T4	1	1	
T5	2	3	
T6	1	3	
T7	1	3	
T8	3	2	
T9	3	1	
T10	3	2	
T11	3	2	
T1	1	2	After Treatment
T3	2	3	
T4	1	1	
T5	2	1	
T6	3	0	
T7	3	1	
T8	1	4	
T9	2	3	
T10	2	3	
T11	3	2	

Supplementary Figure 1. Diseased salmon phenotype (left) and table with the number of healthy and sick fish sampled from each tank (right). The disease manifested itself in the form of large ulcers on the salmon skin and subsequent death of the fish. Not all the fish presented symptoms of the disease. The table on the left shows the number of healthy and sick fish analyzed from each tank. We initially sampled a high number of fish (>80), sampling an equal number of fish from all the replicate thanks. Then we decided to limit our study to 80 fish with equal representativeness of sick and healthy individuals. For this reason, we dropped some of the collected samples which cause the sparse representativeness of the tanks in the analysis (and the absence of tanks 2 and 12). We note that the difference in the number of sick and healthy fish sampled from each tank can be responsible for the observed tank effect.

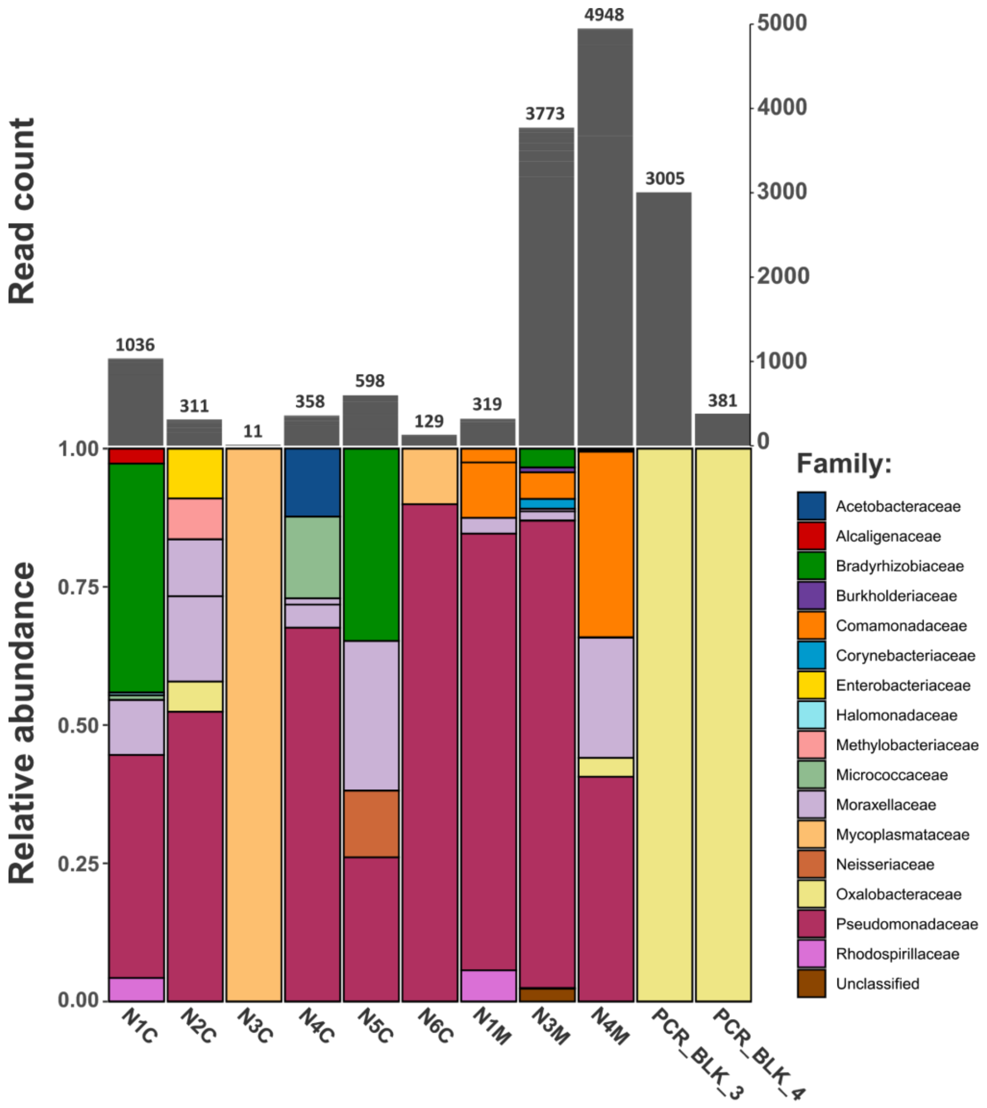

Supplementary Figure 2. DNA extraction and PCR amplification negative controls composition and read count. Relative abundance composition of the DNA extraction negative controls of the gut content samples (N1C, N2C, N3C, N4C, N5C, N6C) and of the gut mucosa samples (N1M, N3M, N4M) and of the negative controls of the PCR amplification (PCR_BLK_3, PCR_BLK_4) (bottom). Putative contaminants are shown at the family taxonomy level. Total read count after the filtering steps for each sample is reported (top). Low numbers of reads were observed for all the negative controls. For comparison, the mean value for a true biological sample is 28007 reads. Pseudomonadaceae (*Pseudomonas* sp.) represent the major DNA extraction contaminant in our dataset. Other relevant DNA extraction contaminants were members of the Bradyrhizobiaceae, Moraxellaceae and Comamonadaceae families. *Ralstonia* (Oxalobacteraceae) was instead recognized to be a PCR reagents contaminant. The Mycoplamsataceae family (*Mycoplasma* sp.) was acknowledged to be a true OTU and its presence here is explained by cross-contamination events between samples during the laboratory handling. Few reads of *Mycoplasma* sp. can be found in the negative controls and only in two out of 11 negative control samples, indicating that it wasn't a major contamination event.

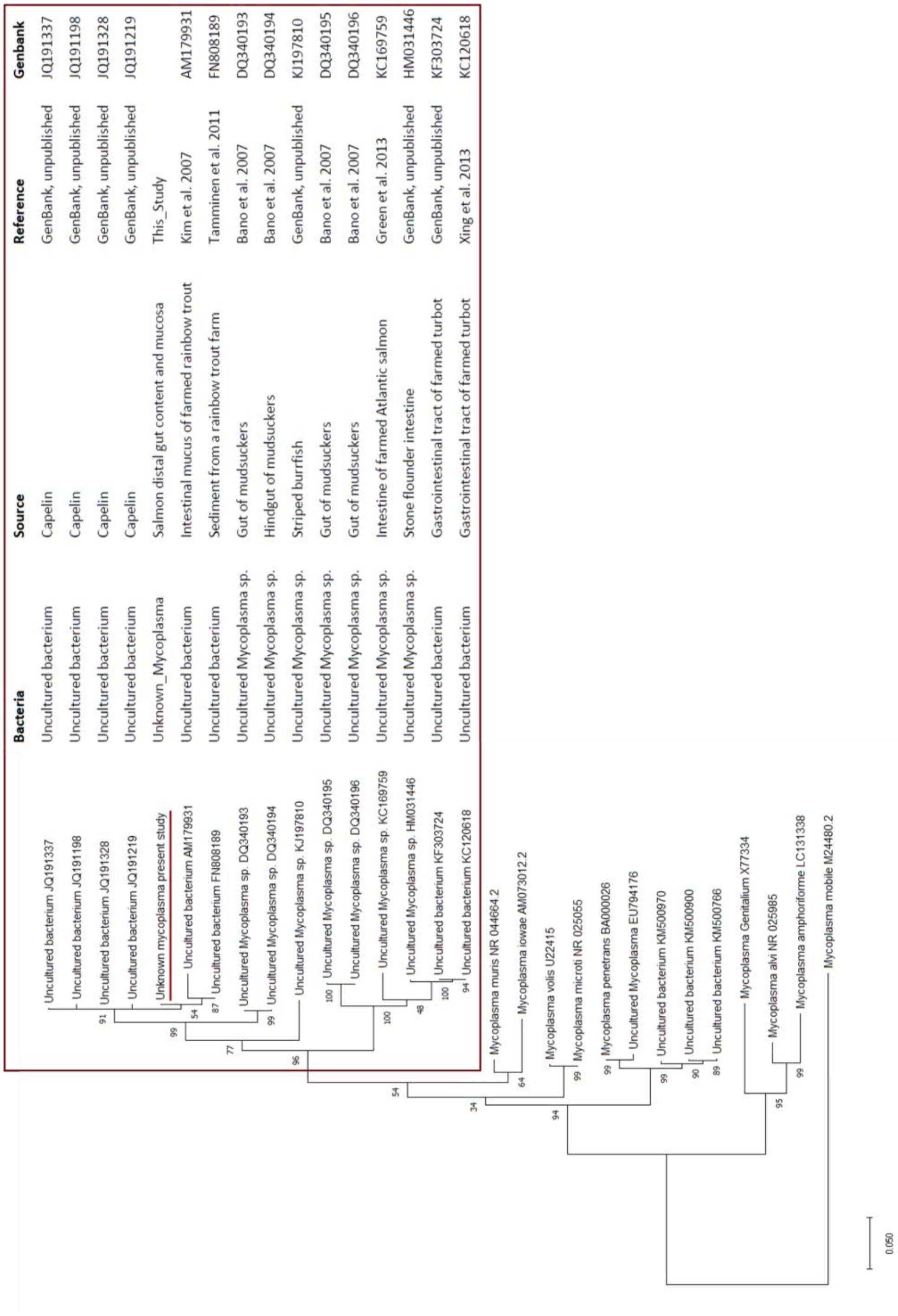

Bacteria	Source	Reference	Genbank
Uncultured bacterium	Capelin	GenBank, unpublished	JQ191337
Uncultured bacterium	Capelin	GenBank, unpublished	JQ191198
Uncultured bacterium	Capelin	GenBank, unpublished	JQ191328
Uncultured bacterium	Capelin	GenBank, unpublished	JQ191219
Unknown_Mycoplasma	Salmon distal gut content and mucosa	This_Study	
Uncultured bacterium	Intestinal mucus of farmed rainbow trout	Kim et al. 2007	AM179931
Uncultured bacterium	Sediment from a rainbow trout farm	Tamminen et al. 2011	FN808189
Uncultured Mycoplasma sp.	Gut of mudsuckers	Bano et al. 2007	DQ340193
Uncultured Mycoplasma sp.	Hindgut of mudsuckers	Bano et al. 2007	DQ340194
Uncultured Mycoplasma sp.	Striped burrfish	GenBank, unpublished	KJ197810
Uncultured Mycoplasma sp.	Gut of mudsuckers	Bano et al. 2007	DQ340195
Uncultured Mycoplasma sp.	Gut of mudsuckers	Bano et al. 2007	DQ340196
Uncultured Mycoplasma sp.	Intestine of farmed Atlantic salmon	Green et al. 2013	KC169759
Uncultured Mycoplasma sp.	Stone flounder intestine	GenBank, unpublished	HM031446
Uncultured bacterium	Gastrointestinal tract of farmed turbot	GenBank, unpublished	KF303724
Uncultured bacterium	Gastrointestinal tract of farmed turbot	Xing et al. 2013	KC120618

Supplementary Figure 3. *Mycoplasma* sp. maximum likelihood phylogenetic tree. A maximum likelihood neighbor-joining phylogenetic tree was built using the *Mycoplasma* sp. sequence and 28 other sequences recovered from NCBI through BLAST search to assess its biological origin. Our sequence cluster with 15 sequences recovered from fish gut or fish-related environments, constituting a coherent clade which separates all the *Mycoplasma* spp. found in fish from those found in other environments.

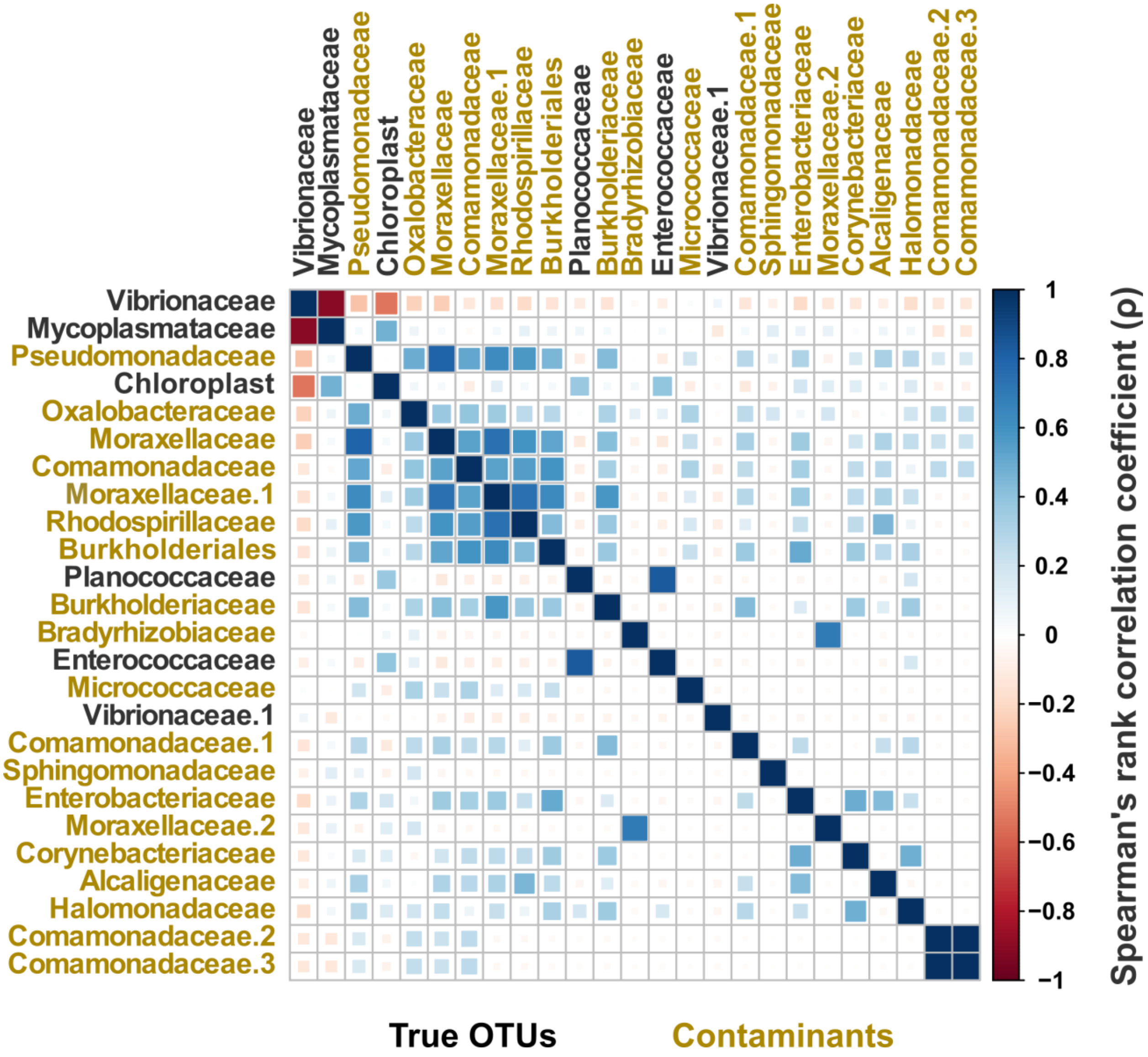

Supplementary Figure 4. OTUs correlation heatmap. Heatmap showing Spearman's correlation coefficient between some OTUs including the most abundant and those regarded as contaminants. Patterns of co-occurrence are characteristic of the reagent contaminants that usually show up together when the true biological signal is weak. Co-occurrence is visible as a positive correlation. OTUs are indicated at the family level when possible and at the order level when the family is unknown. Positive correlations (blue) are visible between the putative contaminants, while negative correlations (red) are visible between contaminants and true OTUs like Vibrionaceae (*Aliivibrio* sp).

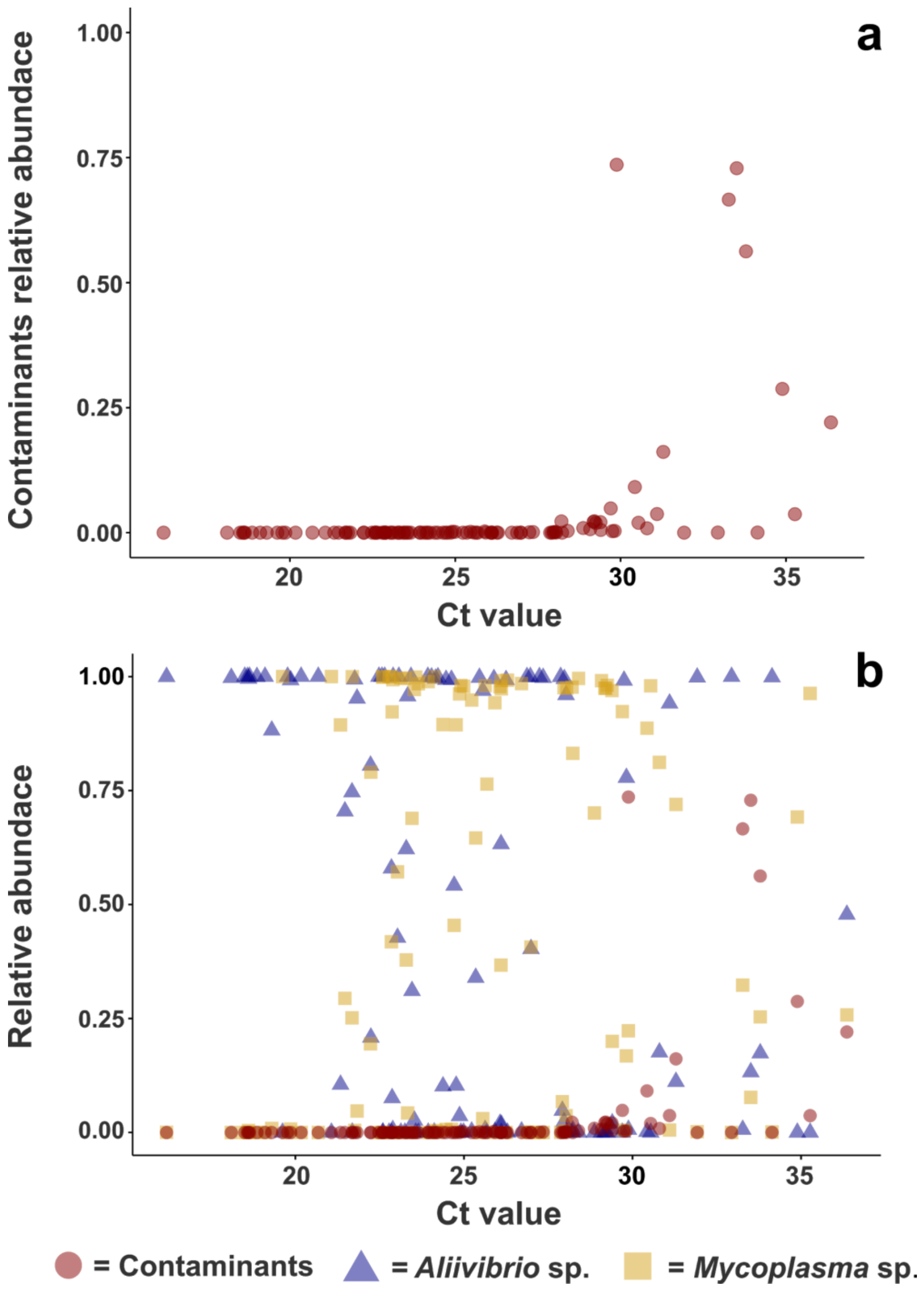

Supplementary Figure 5. Correlations between Ct values and the relative abundance of specific OTUs. When plotting contaminants relative abundances against Ct values (top) it is possible to see that contaminants are insignificant in samples with low Ct values, while increase in relative abundance as the Ct value increases, indicating that contamination is an issue for the samples with lower microbial biomasses. Here the contaminants include all the OTUs identified in the negative controls excluding *Mycoplasma* sp. For comparison contaminans were plotted together with OTUs recognized as genuine: *Aliivibrio* sp. and *Mycoplasma* sp. (bottom). *Aliivibrio* and *Mycoplasma* showed to characterize samples with lower Ct values.

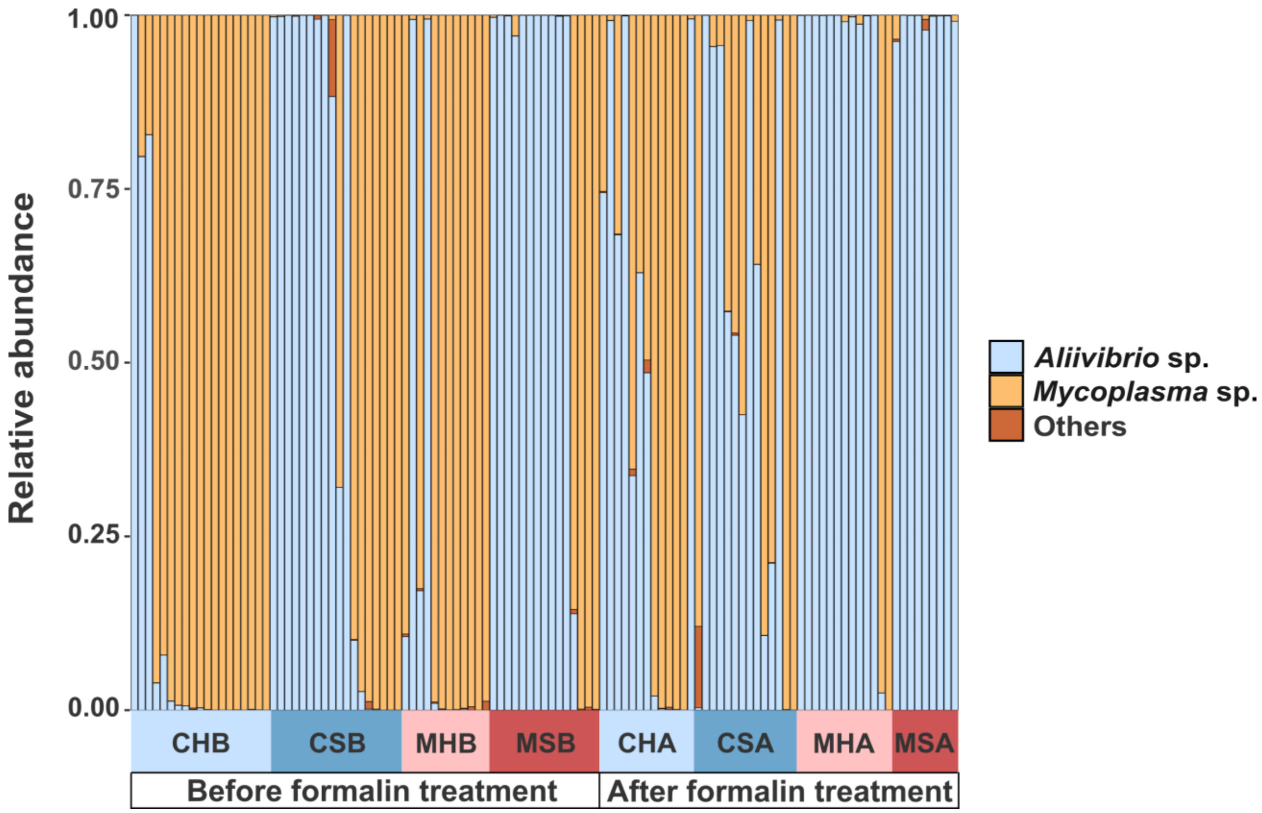

Supplementary Figure 6. Microbial composition of the individual samples plotted according to the group of origin. The composition of the individual samples shows that most of the samples were dominated by just one OTU.

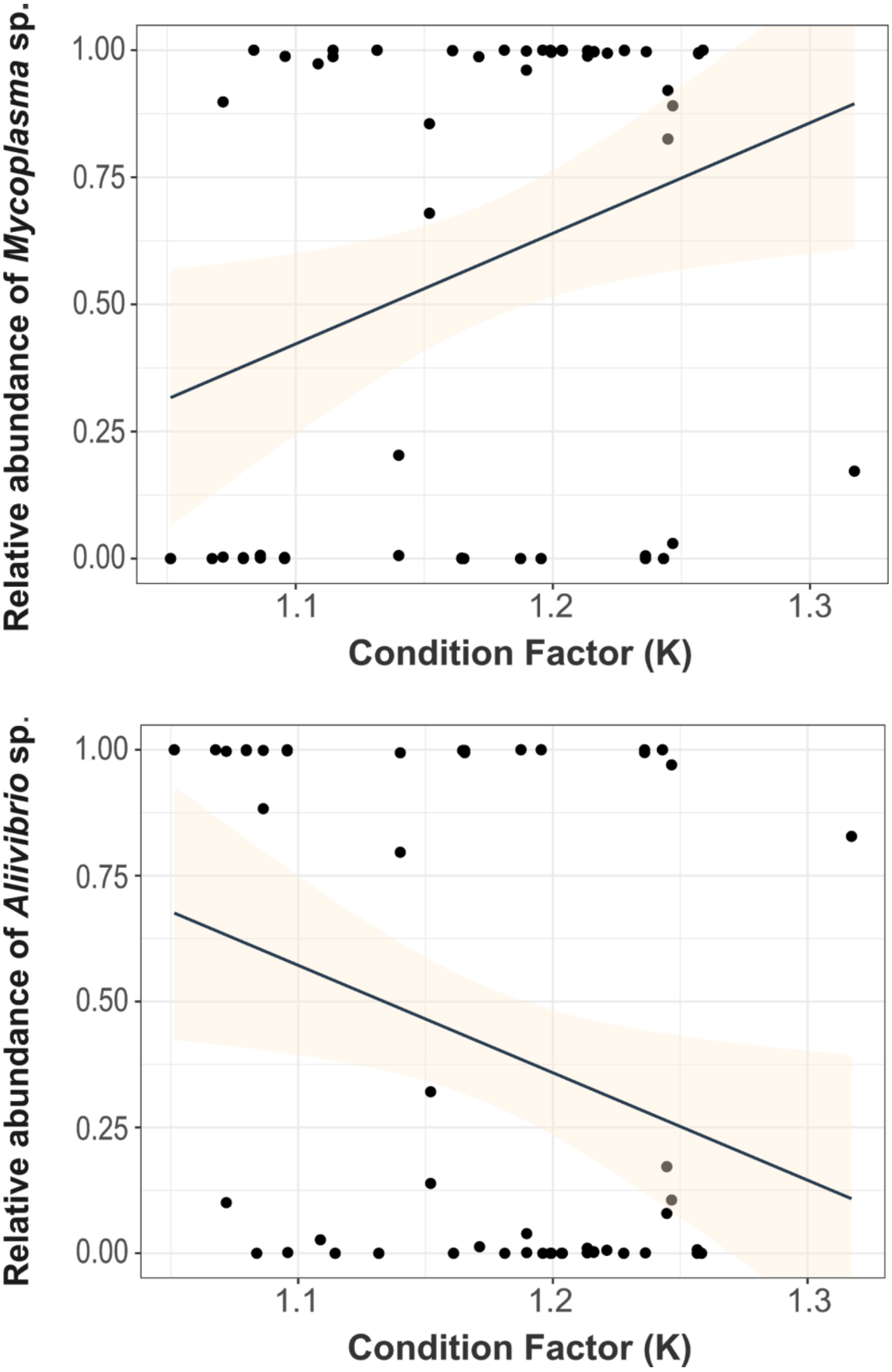

Supplementary Figure 7. Spearman's rank correlation between OTUs relative abundance and condition factor K. A pattern similar to that observed with fish weight was observed for condition factor K, although with less strong correlations. A positive correlation (Spearman's R = 0.27, $p < 0.05$) was found when comparing *Mycoplasma* sp. relative abundance and fish condition factor K (top) and a negative correlation (Spearman's R = -0.26, $p < 0.05$) was observed when comparing *Aliivibrio* sp. relative abundance with fish condition factor K.

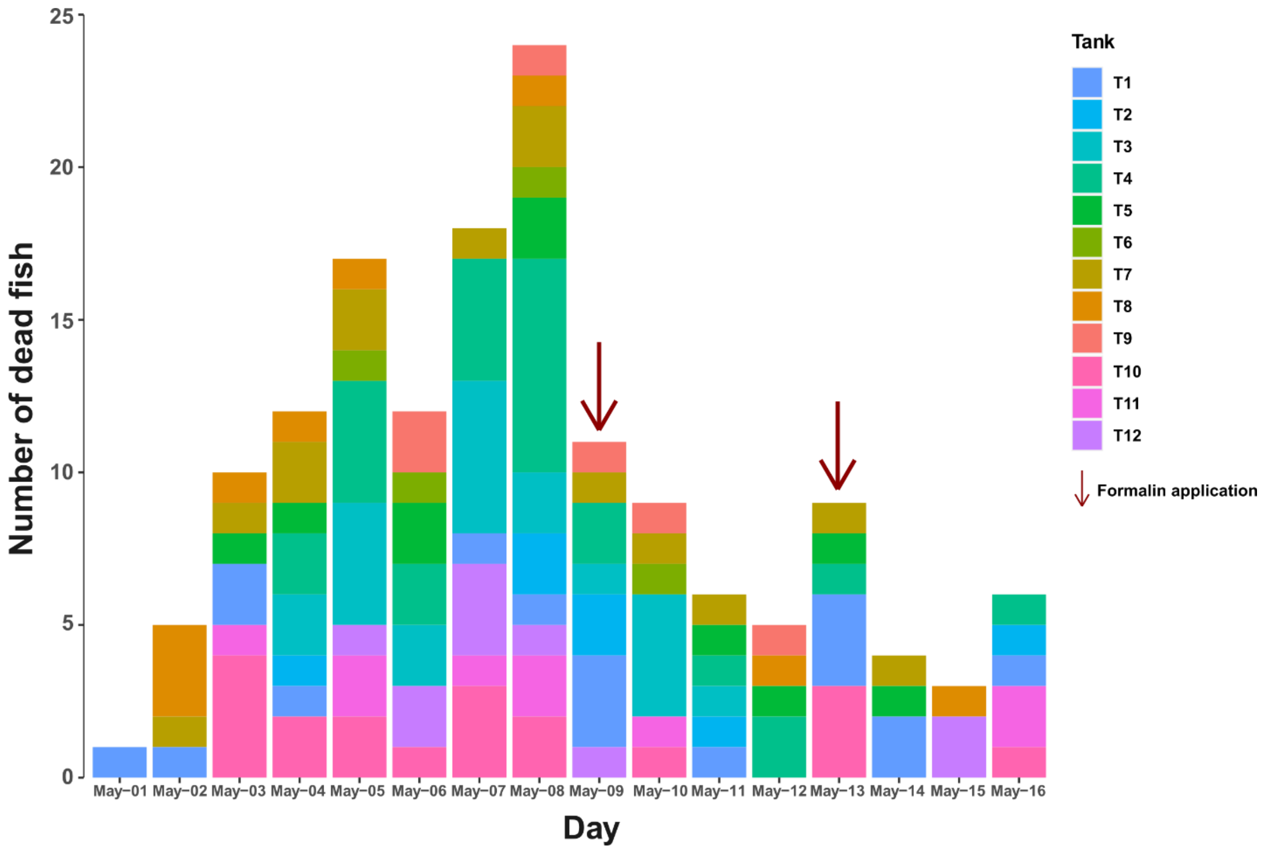

Supplementary Figure 8. Dead fish count. Dead fish count for each day of the trial starting from May-01-2019, day in which the first death caused by the infection was observed, until May-16-2019, day in which the trial was stopped after the second sampling. Formalin application days are indicated with arrows. Colors identify the tanks in which the dead fish were found.
