# Supplementary material for: Salmon gut microbiota correlates with disease infection status: potential for monitoring health in farmed animals
Source: Anim Microbiome. 2021 Apr 20;3:30. doi: 10.1186/s42523-021-00096-2 (PMC8056536; doi:10.1186/s42523-021-00096-2)
Supplement: Supplementary file 3 — Additional file 3. DNA extraction protocol. [file 42523_2021_96_MOESM3_ESM.pdf]

Additional file 3

DNA extraction protocol

DNA extraction reagent composition (for 50 mL of each buffer):

DNA Binding buffer, 50 mL		Amount	Unit	Final concentration	Unit
	GuSCN	29.5	gr	5	M
	Citrate buffer, pH 5	5	ml	10	mM
	n-lauryl sarcosine	2.5	ml	1	%
	ddH2O (to 50 ml)				
Wash Buffer A, 50 mL					
	GuSCN	11.8	gr	2	M
	Isopropanol	30	ml	60	%
	Citrate buffer, pH 5	5	ml	10	mM
	Tween-20	50	ul	0,05	%
	ddH2O (to 50 ml)				

Washing Buffer B, 50 ml					
	Absolute Ethanol	40	ml	80	%
	ddH2O	10	ml		

The protocol was followed for both distal gut content and distal gut mucosa samples.

Preparation of buffers

Prior to the extraction, G-beads were washed and premixed with Binding buffer (buffer B): Briefly,1.5 ml of silica magnetic beads (G-BIOSCIENCES®) were transferred to a 2 ml Eppendorf tube. The tube was placed on a magnetic rack and the supernatant removed. Beads were washed twice in 2 ml of TE buffer and then added to 20 ml of buffer B (15 µl of beads + 200 µl binding buffer B for each individual sample). The pre-mixed solution containing buffer B + beads was stored in the fridge, protected from light and then re-equilibrated at room temperature before use.

Extraction procedure:

Selected samples stored in 1x DNA/RNA Shield™ buffer (Zymo Research) were thawed and vortexed a few seconds, followed by bead beating, using a TissueLyser (Qiagen) for 3 minutes at 30Hz. After cell lysis, samples were centrifuged at 17000xg for 1 minute. For each lysed sample, 200 µl of supernatant were transferred to a new Eppendorf tube and 500 ul were stored in another tube as a backup and frozen at -20°C.

200 µl of buffer B + beads were added to each sample and incubated on a thermal shaker for 15 minutes at 10°C, 1500 rpm. A negative control was included for each day of extraction to check

for contamination. For the negative control, 200 µl of buffer B + beads were mixed with 200 µl of 1x DNA/RNA Shield buffer, the original buffer in which the samples had been conserved.

After the incubation, samples were shortly spun down and placed on a magnetic rack. Beads were allowed to settle for 1 minute and the supernatant subsequently removed and discarded. Tubes were removed from the magnet and the DNA containing beads were washed in 200 µl of buffer C, followed by two washes in 80% ethanol to remove salts. After the final ethanol wash the beads were allowed to air-dry (on the magnet) for 5 minutes.

After 5 minutes of evaporation, 50 µl of EB elution buffer (10 mM Tris-HCl) were added to each sample. Tubes were incubated for 5 minutes at 25°C (off magnet), at 1400 rpm before being returned to the magnet. Beads were allowed to settle and the DNA containing supernatant transferred to a new Eppendorf Lo-Bind 1.5 ml tube. Samples were stored at -20°C until further downstream processing.
